# Quantitative Porosity Engineering of Carbon Electrode in Lithium–Oxygen Batteries with Cell‐Level Gravimetric Energy Density Over 1500 Wh kg^−1^

**DOI:** 10.1002/advs.202514406

**Published:** 2025-10-15

**Authors:** Arghya Dutta, Takashi Kameda, Junji Takada, Yuuka Nakajima, Takahiro Morishita, Shoichi Matsuda

**Affiliations:** ^1^ Center for Green Research on Energy and Environmental Materials National Institute for Material Science 1‐1 Namiki Tsukuba Ibaraki 305‐0044 Japan; ^2^ Kondo Teruhisa Memorial Advanced Carbon Technology Center Toyo Tanso Co., Ltd. 5‐7‐12 Takeshima Nishiyodogawa‐ku Osaka Japan 555‐0011; ^3^ Center for Advanced Battery Collaboration National Institute for Material Science 1‐1 Namiki Tsukuba Ibaraki 305‐0044 Japan

**Keywords:** carbon electrode, high energy battery, lean electrolyte, Lithium‐oxygen battery, porosity optimization

## Abstract

Lithium–oxygen batteries (LOBs) offer an extremely high theoretical energy density; however, their practical realization depends strongly on the design of porous carbon positive electrodes. Most prior efforts have emphasized material design while overlooking the role of the electrolyte stored within pores, leaving the design principles for achieving practical high‐energy‐density LOBs unclear. In the present study, through simulations, it is quantitatively demonstrated that while increasing pore volume initially improves energy density, it eventually plateaus due to increasing electrolyte demand. The simulations indicate that reduced electrolyte volumes and optimized mass loading of the positive electrode are crucial for maximizing energy density. Experimental validation with systematically tuned carbon electrodes in pouch‐type LOBs with realistic mass‐loadings supports these findings. While large pore volumes enhance capacity, they require excessive electrolyte, ultimately counter‐balancing energy density. Conversely, lowering electrolyte volumes in highly porous electrodes leads to incomplete filling, increased impedance, enhanced parasitic reactions, and poor cycling stability. As a result, by tailoring the pore structure, electrodes capable of delivering cell‐level energy density exceeding 1500 Wh kg^−1^ and maintaining stable cycling under capacity‐limited conditions are demonstrated. This work redefines the role of pore engineering in LOB electrodes, highlighting its crucial contribution to achieving practical, high‐energy, and long‐lasting LOBs.

## Graphical abstract

While large pore volumes in carbon electrodes are believed to boost the energy density of lithium‐oxygen batteries, simulations and experiments show that excessive porosity increases electrolyte demand, reducing net energy gains. Optimizing pore structure for lean electrolyte operation enables >1500 Wh kg^−1^ pouch cells with good stability, redefining pore engineering as a key strategy for practical lithium–oxygen battery development.

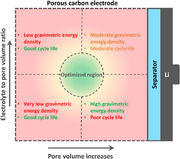

## 1 Introduction

Rechargeable lithium–oxygen (Li–O_2_) batteries (LOBs) are widely recognized as a promising next‐generation energy storage technology, with theoretical gravimetric energy densities surpassing those of conventional lithium‐ion batteries (LIBs).^[^ ^1^, ^2^ ^]^ While the commercial positive electrode materials in LIBs offer gravimetric energy densities of 500–800 Wh kg^−1^, LOBs can theoretically achieve ≈3500 Wh kg^−1^ when evaluated solely based on the redox‐active material.^[^ ^3^ ^]^ Despite this potential, practical implementation remains hampered by several critical challenges, particularly the design and optimization of the porous carbon positive electrode.^[^ ^4^, ^5^, ^6^ ^]^ In nonaqueous LOBs, the formation of insoluble lithium peroxide (Li_2_O_2_) during discharge obstructs oxygen and lithium‐ion pathways, significantly restricting the effective utilization of the porous carbon electrode.^[^ ^7^ ^]^ Extensive research has focused on how the structural features of carbon electrodes, such as surface area, pore size, and pore volume, influence their discharge performance.^[^ ^8^, ^9^, ^10^, ^11^, ^12^, ^13^, ^14^ ^]^ For instance, Meini et al. demonstrated that electrodes with larger carbon surface areas typically deliver enhanced discharge capacities.^[^ ^8^ ^]^ Ding et al. examined diverse carbon materials, including carbon blacks, mesoporous carbons, multiwalled carbon nanotubes, and reduced graphene oxide, revealing a clear positive correlation between pore size and discharge capacity.^[^ ^9^ ^]^ In contrast, Kuboki et al. underscored the importance of electrode pore volume, showing a stronger link between this parameter and discharge performance.^[^ ^10^ ^]^ Despite these differing emphases, there is broad consensus that an optimal air electrode for LOBs should combine three essential characteristics: (i) high surface area to support electrochemical activities and accommodate discharge product deposition, (ii) sufficiently large‐sized pores to promote effective oxygen and lithium‐ion transport and avoid pore blockage, and (iii) high pore volume to provide ample space for the growth of discharge products such as Li_2_O_2_.

Although a lot of work has been done with the emphasis on increasing the electrode porosity, the optimization of electrolyte volume used in a cell has, in general, remained an overlooked factor in LOB research.^[^ ^15^, ^16^, ^17^ ^]^ Typically, LOBs have been evaluated using an excess of electrolyte (>50 µL cm^−2^), low areal loading of carbon material, and limited areal capacity (<1 mAh cm^−2^).^[^ ^18^, ^19^ ^]^ This practice not only limits the attainable cell‐level energy density, often yielding values lower than those of conventional LIBs, but also obscures a comprehensive analysis by artificially inflating performance metrics. Our recent work highlights the relevance of the electrolyte‐to‐capacity (E/C) ratio, a metric traditionally used in LIBs, as a valuable descriptor for assessing LOB energy density.^[^ ^20^ ^]^ Specifically, we find that maintaining an E/C ratio below 10 g Ah^−1^ is essential for achieving practically relevant gravimetric energy densities in LOBs. Moreover, given that the electrolyte accounts for a substantial fraction of the total LOB cell cost, reducing its content also yields a significant economic advantage. In addition to reducing the electrolyte volume, high gravimetric energy density requires positive electrodes with substantial mass loadings (>4 mg cm^−2^) and high areal capacities (>4 mAh cm^−2^). However, increasing the electrode thickness exacerbates pore clogging by discharge products, leading to underutilized active material and emphasizing the critical role of high porosity in ensuring full electrode utilization.^[^ ^21^ ^]^ Yet, high porosity introduces a key trade‐off: although it enhances discharge capacity, it also demands a larger electrolyte volume to ensure complete pore filling and adequate ionic transport within the structure, which can reduce cell‐level gravimetric energy density. Despite this understanding, systematic and quantitative studies addressing how to engineer and optimize the porous structure of carbon‐based positive electrodes for practical high‐energy LOBs operating under lean electrolyte conditions remain scarce. Addressing this gap is crucial for realizing the full potential of LOBs, achieving high gravimetric energy density, and ensuring stable cycling under practical conditions.

This study aims to quantitatively evaluate the impact of electrode porosity on electrolyte demand and gravimetric energy density in LOBs and to strategically engineer porosity to enable both high gravimetric energy density and extended cycle life under lean electrolyte conditions. Through simulations, we first clarify the critical trade‐off: while increasing pore volume initially boosts gravimetric energy density, this benefit gradually plateaus as electrolyte requirements also increase. We then experimentally validate this trade‐off using a series of systematically engineered porous carbon materials with controlled variations in surface area, pore diameter, and pore volume. These electrodes, with practical carbon loadings exceeding 4.0 mg cm^−2^, were integrated into pouch‐type LOBs and evaluated under lean electrolyte conditions. Detailed analyses show that although larger pore diameters and volumes enhance absolute capacity, they do not necessarily yield higher specific capacity or gravimetric energy density at the same rate due to increased electrolyte demand. Conversely, in attempts to lower the amount of electrolyte, cells with high pore diameters and volumes suffered from incomplete filling, electrolyte depletion, increased impedance, voltage polarization, and accelerated electrochemical degradation. These findings underscore the importance of precise pore structure optimization to balance electrolyte use and energy density. By tailoring the pore structure, we demonstrate electrodes capable of delivering gravimetric energy densities exceeding 1500 Wh kg^−1^ (normalized to total cell mass, including electrolyte), maintaining stable cycling in capacity‐limited conditions. This work redefines the conventional understanding that higher porosity universally enhances LOB performance, highlighting the crucial role of optimized porosity in achieving practical, high‐energy LOB systems.

## 2 Results

### 2.1 Simulation of Gravimetric Energy Density Variation with Pore Volume

**Figure 1:**
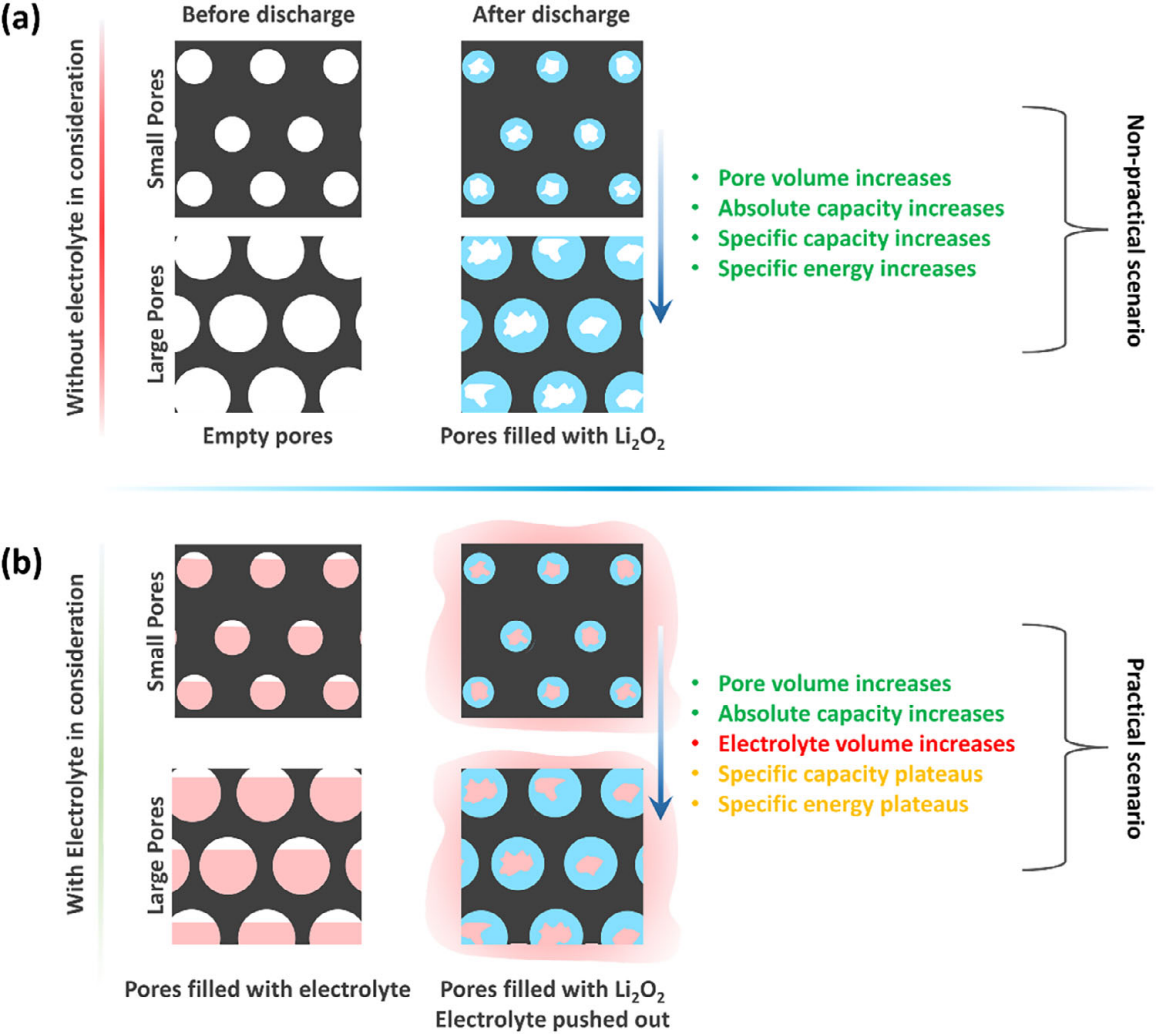
Schematic illustrations of Li_2_O_2_ deposition (discharge process) inside the electrode pores a) without electrolyte and b) with electrolyte in consideration.

Since solid Li_2_O_2_ is deposited during the discharge process of LOBs, it is intuitive that carbon materials with wide pore diameters, large pore volumes, and high surface areas, providing ample space for accommodating Li_2_O_2_, would result in higher cell capacities. Typically, the specific capacity of LOBs is normalized only to the mass of the carbon material, often neglecting the contribution of electrolyte mass.^[^ ^9^, ^16^, ^18^, ^19^, ^22^ ^]^ Consequently, both the specific capacity and gravimetric energy density of the cell tend to increase with pore volume, as illustrated schematically in **Figure**  **1**a. However, in practical applications, the electrolyte represents the largest mass fraction among all cell components.^[^ ^20^ ^]^ Therefore, considering the mass of the electrolyte is crucial when developing high‐energy‐density, practically viable cells. While high porosity in the carbon material is advantageous for increasing the absolute capacity, it also necessitates a larger amount of electrolyte to fill the pores.

Figure 1b schematically illustrates that as pore volume increases, the required electrolyte volume correspondingly increases. This added electrolyte mass offsets the capacity gains, suggesting that the positive effect of high porosity on specific capacity and gravimetric energy density is expected to be constrained.

**Figure 2:**
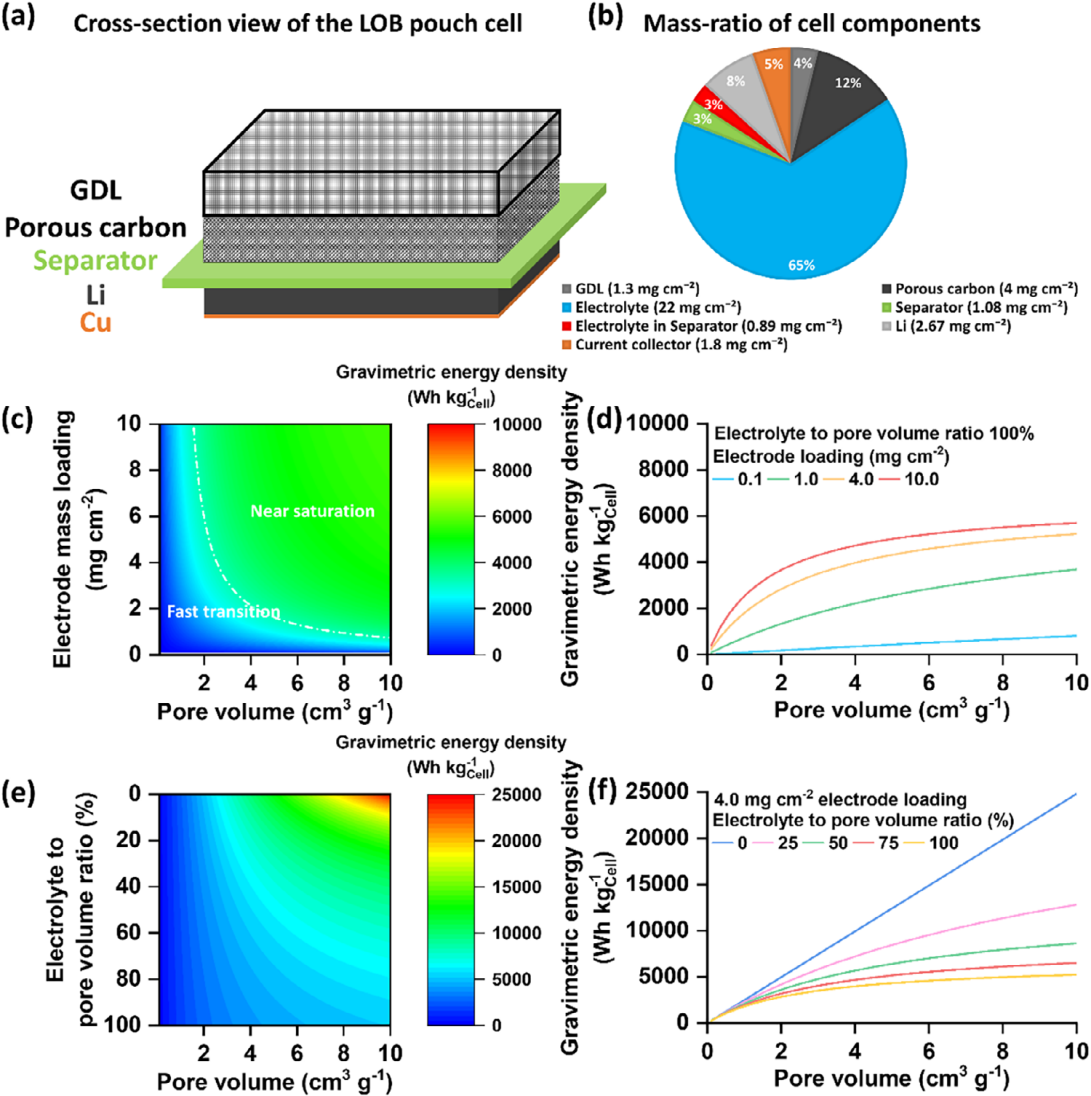
a) Schematic representation of the cross‐section and b) mass ratio of different cell components of the LOB pouch cell employed in this study. c) Contour map of the simulated gravimetric energy density (total cell mass) against pore volume and electrode mass loading, with 100% of the pores filled with electrolyte. d) Line plots of simulated gravimetric energy density against pore volume for four specific electrode loading conditions. e) Contour map of the simulated gravimetric energy density (total cell mass) against pore volume and electrolyte loading, for an electrode loading of 4 mg cm^−2^. f) Line plots of simulated gravimetric energy density against pore volume for five electrolyte loading conditions.

Next, we have conducted simulations to quantitatively assess the effects of electrode porosity and electrolyte loading on the gravimetric energy density of a pouch‐type LOB cell. The cell configuration and the mass distribution of the cell components under consideration are shown in **Figure** **2**a,b. The details of the simulation are provided in the Note S1 (Supporting Information). A practically relevant electrode loading of 4.0 mg cm^−2^ was selected, with the assumption that the electrode pores are fully saturated with electrolyte (100% filling). The mass of all other cell components, including the electrolyte, was also included in the gravimetric energy density calculation, and the average discharge voltage was set at 2.7 V versus Li/Li⁺. While high electrode loading is generally considered advantageous for achieving higher gravimetric energy density, a comprehensive quantitative assessment that accounts for both pore volume and electrolyte mass has been lacking. We first examined how electrode loading (0.1–10 mg cm^−2^) and pore volume (0–10 cm^3^ g^−1^) simultaneously influence the gravimetric energy density. Figure 2c presents a contour map of gravimetric energy density as a function of these two parameters, under the assumption of complete pore filling with Li_2_O_2_ and 100% electrolyte saturation. The results show that increasing both pore volume and electrode loading initially leads to a sharp rise in gravimetric energy density, reaching ≈3000 Wh kg^−1^ at a pore volume of 3 cm^3^ g^−1^ and an electrode loading of 2.5 mg cm^−2^. However, beyond these values, the increase in gravimetric energy density slows significantly. This trend is further clarified in Figure 2d, which shows line plots of gravimetric energy density versus pore volume at various electrode loadings. Increasing loading from 0.1 to 1.0 mg cm^−2^ yields a substantial improvement in gravimetric energy density; for example, at a pore volume of 4 cm^3^ g^−1^, the gravimetric energy density rises from ≈400 to ≈2200 Wh kg^−1^. A further increase to 4.0 mg cm^−2^ boosts the value to ≈4000 Wh kg^−1^. However, raising the loading to 10 mg cm^−2^ results in only a modest gain (≈4600 Wh kg^−1^), suggesting that excessive electrode mass‐loading does not substantially enhance energy metrics. A similar analysis with the electrode thickness and porosity, in Figure S1a,b (Supporting Information), shows the same trend. Increasing electrode thickness does not linearly improve gravimetric energy density; instead, thick electrodes often face mass transport limitations, uneven Li_2_O_2_ deposition, and incomplete utilization, resulting in lower‐than‐expected gravimetric energy density.^[^ ^21^ ^]^ Based on these insights, we fixed the electrode loading at 4.0 mg cm^−2^ and systematically varied electrolyte loading to investigate its influence on gravimetric energy density. Figure 2e shows a contour plot depicting the relationship between gravimetric energy density and electrode pore volume at various electrolyte‐loading levels. The analysis assumes that the entire pore volume is initially occupied by electrolyte and is subsequently filled by Li_2_O_2_ upon discharge, with full pore utilization for product deposition.

The data indicate that the impact of pore volume on gravimetric energy density is highly dependent on the amount of electrolyte. When the electrolyte loading exceeds 80%, the gravimetric energy density increases sharply with pore volume up to ≈4 cm^3^ g^−1^, beyond which further gains are minimal. This suggests that under high electrolyte‐loading conditions, the advantage of increasing pore volume is quickly saturated. On the other hand, reducing the electrolyte content has a much stronger influence on enhancing the gravimetric energy density. Figure 2f simplifies these observations: at 100% pore filling, increasing the pore volume from 4 to 10 cm^3^ g^−1^ raises the gravimetric energy density from ≈4000 to only ≈5100 Wh kg^−1^, a modest 27% gain. In contrast, reducing electrolyte loading to 75%, 50%, and 25% of the pore volume at 10 cm^3^ g^−1^ leads to dramatic increases in gravimetric energy density, reaching ≈6600, 8700, and 12900 Wh kg^−1^, respectively. The corresponding contour plot and line plots for electrolyte loading and porosity of the electrode are shown in Figure S2a,b (Supporting Information).

Taken together, these findings demonstrate that while increasing pore volume can initially boost specific capacity and gravimetric energy density, the benefits saturate beyond a certain threshold. Instead, minimizing the electrolyte volume and properly optimizing the mass‐loading/thickness of the electrode emerge as essential parameters to maximize gravimetric energy density. Nevertheless, operating under lean electrolyte conditions with large‐pore electrodes introduces additional complexities, such as incomplete electrode filling, electrolyte depletion, and long‐term stability challenges, all of which must be carefully addressed.^[^ ^20^, ^23^ ^]^ In the following sections, we present a systematic approach to engineer porous carbon materials with controlled pore structures and investigate how this optimization of electrode architecture and electrolyte volume governs both the gravimetric energy density and cycling stability of LOBs, critical factors for translating this technology into practical, high‐energy applications.

### 2.2 Synthesis of MgO Templated Porous Carbon Powders

Nanoporous carbon materials were synthesized through a hard‐templating approach, employing phenol resin as the carbon source and magnesium oxide (MgO) as the hard template. This methodology affords precise control over the resulting mesopore size of the carbon materials, which can be tailored by adjusting the dimensions of the MgO template. The synthesis procedure involved subjecting a mixture of phenol resin and MgO to heat treatment at 900 °C under nitrogen (N_2_) atmosphere, leading to the carbonization of the precursor material. Subsequently, the selective dissolution of the MgO template was carried out at room temperature using diluted sulfuric acid. This step resulted in the isolation of the carbon structure, followed by a secondary heat treatment at 1800 °C. The resultant mesoporous carbons, characterized by varying pore diameters, are identified as MPC‐5, MPC‐10, MPC‐18, MPC‐33, and MPC‐38, with each number indicating the respective pore diameter in nanometers. The synthesis process of the carbons is schematically shown in Figure S3 (Supporting Information).

### 2.3 Physico‐Chemical Characterization of the Carbon Materials

**Figure 3:**
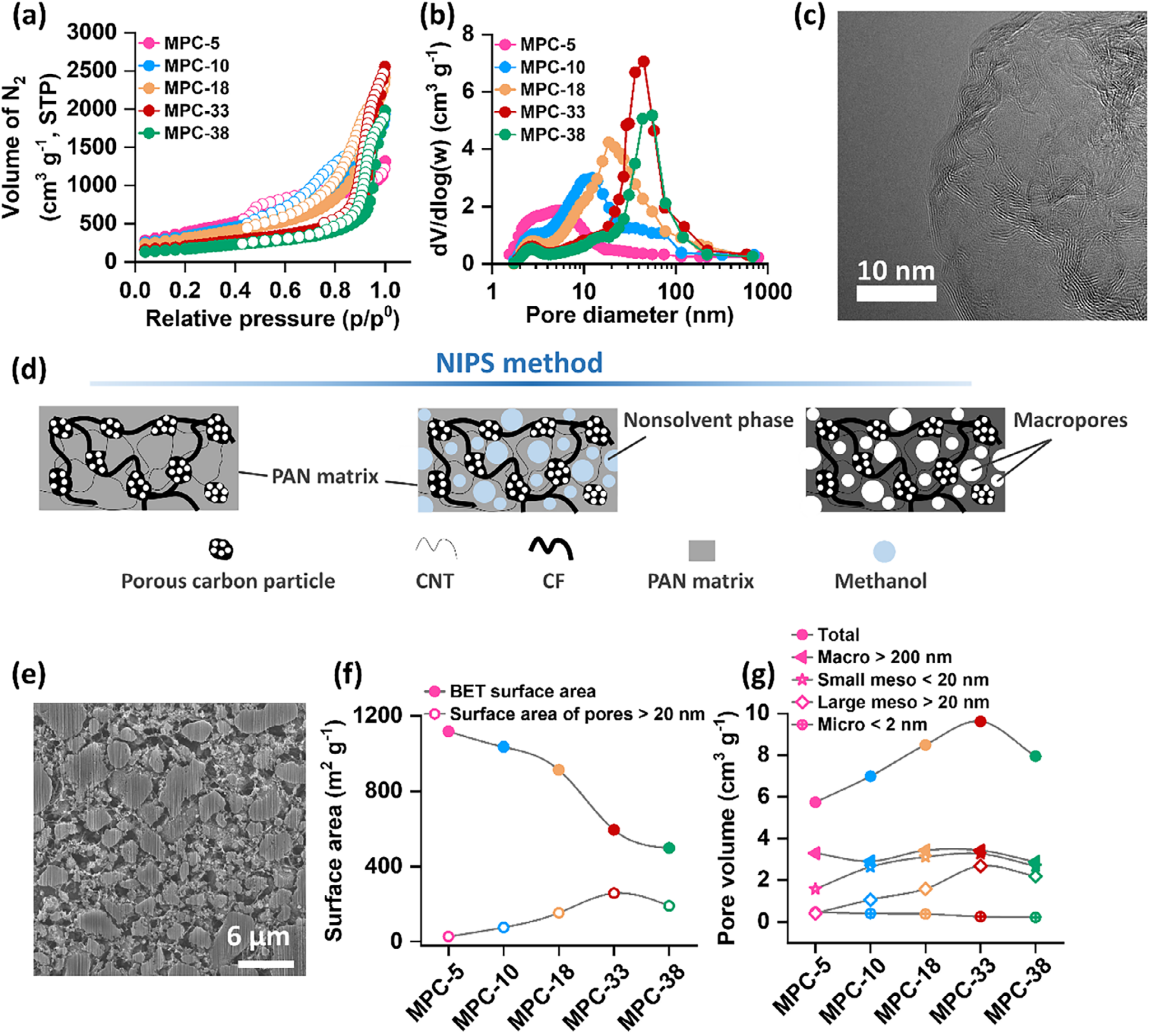
a) N_2_ adsorption/desorption isotherms and b) BJH pore size distribution (from adsorption data) of different carbon powders. c) TEM image of the MPC‐10 powder sample. d) Schematic illustration of the NIPS method employed to fabricate the porous carbon electrodes. e) Cross‐sectional SEM image of the MPC‐10 electrode. Comparison of f) surface area and g) pore volume of different porous carbon electrodes.

A comprehensive investigation into the physicochemical properties of the porous carbon materials was conducted using various analytical techniques. N_2_ adsorption and desorption measurements at ‐196 °C were utilized to determine the surface area, pore volume, and pore diameter of the powder samples. The N_2_ adsorption/desorption isotherms and the Barrett‐Joyner‐Halenda (BJH) pore size distribution curves are depicted in **Figure** **3**a,b, respectively, with summarized results provided in Table S1 (Supporting Information). The analysis reveals that the samples exhibited a range of pore diameters from 5 to 38 nm. Interestingly, there was a gradual decrease in the BET (Brunauer−Emmett−Teller) surface area of the samples as the pore diameter increased, following this sequence: MPC‐5 (1423 m^2^ g^−1^) > MPC‐10 (1233 m^2^ g^−1^) > MPC‐18 (1117 m^2^ g^−1^) > MPC‐33 (757 m^2^ g^−1^) > MPC‐38 (623 m^2^ g^−1^). However, the pore volume generally showed an increasing trend with the enlargement of the pore diameter, except for MPC‐38. The trend of pore volume follows this sequence: MPC‐5 (2.0 cm^3^ g^−1^) < MPC‐10 (2.98 cm^3^ g^−1^) < MPC‐38 (3.04 cm^3^ g^−1^) < MPC‐18 (3.71 cm^3^ g^−1^) < MPC‐33 (3.93 cm^3^ g^−1^). We qualitatively examined the oxygen‐containing functional groups using X‐ray photoelectron spectroscopy (XPS), focusing on MPC‐10 as a representative case. The XPS C1s spectrum (Figure S4, Supporting Information) reveals the presence of various functional groups. In addition to the components at 284.6 and 285.5 eV corresponding to sp^2^ and sp^3^ hybrid forms of carbon, we observed C−O functional groups, including phenol, ether, and epoxy, with binding energies ≈286.5 eV.^[^ ^24^ ^]^ Furthermore, C═O functional groups, such as carbonyl and quinone, were detected at ≈287.5 eV, and carboxyl carbon at 288.9 eV.^[^ ^24^ ^]^ The corresponding peaks for these functional groups in the O1s spectrum are shown in Figure S5 (Supporting Information).

Concerning microstructural characterization, scanning electron micrographs (SEM) in Figure S6 (Supporting Information) illustrate that at lower magnifications, all carbon samples display a particle‐like morphology, with sizes distributed across several micrometers. However, upon closer examination at higher magnification (as depicted in Figure S7, Supporting Information), it becomes apparent that these particles are comprised of interwoven carbon flakes. Figure S8 (Supporting Information) illustrates the Raman spectrum of MPC‐10, serving as a representative material and offering significant insights into the structural properties of carbon. The presence of the D band at 1350 cm^−1^ signifies vibrations associated with sp^3^‐bonded carbon atoms or defects, while the G band at 1580 cm^−1^ suggests the presence of sp^2^‐bonded carbon atoms within a more graphitized carbon framework.^[^ ^25^ ^]^ Furthermore, the I_D_/I_G_ intensity ratio of 1.49 indicates a turbostratic structure for the carbon. Figure S9 (Supporting Information) depicts the X‐ray diffraction (XRD) pattern of carbon sample MPC‐10, indicating the development of a graphitic structure. The XRD patterns of the carbons show a prominent diffraction peak at 2θ  =  24°, corresponding to the (002) crystallographic plane of graphite crystallites. Another peak at ≈43° is considered as an overlap of two peaks. The peak at 42.4°, corresponding to the (100) plane, while the other peak at 44.3°, is associated with the (101) plane.^[^ ^26^ ^]^ The randomly stacked graphitic layers in the carbon material MPC‐10 are visually confirmed through the high‐resolution transmission electron micrograph (TEM) shown in Figure 3c. The appearance of graphitic layers provides direct evidence of graphitization in the carbon. Additionally, the TEM image confirms the presence of mesoporosity in the sample.

### 2.4 Fabrication of Self‐Standing Porous Carbon Membrane

The synthesized carbon materials were employed in the fabrication of a self‐standing porous carbon membrane using a technique known as nonsolvent‐induced phase separation (NIPS).^[^ ^23^ ^]^ In this process, a solvent that does not dissolve the polymer is introduced into a polymeric film, leading to the creation of interconnected voids.^[^ ^27^ ^]^ This ultimately results in the formation of a sponge‐like structure within the polymer film. By employing this methodology, we successfully produced carbon membranes featuring a hierarchical macro‐mesoporous interconnected network, which is expected to enhance both the transport properties and capacity of the electrode. To create these self‐standing membranes, we combined the synthesized porous carbon powders with carbon fibers (CF), carbon nanotubes (CNTs), and polymeric materials. The incorporation of CF and CNTs served to enhance the mechanical strength of the membrane in preventing the disintegration of the electrode during cycling. We prepared a typical slurry mixture comprising 75 wt.% carbon powder, 5 wt.% CF, 5 wt.% CNTs, and 15 wt.% PAN (polyacrylonitrile). This slurry was utilized to generate a uniform carbon film using a wet film‐forming method involving a doctor blade. Subsequently, the film was immersed in methanol, acting as a poor solvent, to induce the formation of a porous film through the NIPS process. The resulting membranes were subsequently dried and subjected to an appropriate heat treatment, followed by carbonization at 1050 °C within N_2_ atmosphere. Figure 3d displays a schematic illustration of the fabrication of the hierarchical porous carbon membrane using the NIPS method. The cross‐section of the membrane prepared with MPC‐10 as a representative was observed using a focus ion beam scanning electron microscopy (FIB‐SEM). The micrograph in Figure 3e clearly demonstrates the interparticle macroporosity in the membrane. This SEM analysis complements the observed mesoporosity in the TEM, confirming a hierarchical porous structure of the membrane.

### 2.5 Analysis of the Porosity of the Self‐Standing Carbon Membrane

To characterize the surface area, pore volume, and pore diameter of the membranes, N_2_ adsorption and desorption measurements were performed at ‐196 °C. The resulting N_2_ adsorption/desorption isotherms and the pore size distribution curves, analyzed using the BJH method, are depicted in Figures S10 and S11 (Supporting Information), respectively. Examination of the pore size distribution curves indicates that the carbon membranes maintain pore sizes similar to those of the powder samples. However, it is noted that the surface area and pore volume of the membranes are slightly lower compared to the powders, which can be attributed to the incorporation of CNT, carbon fibers, and PAN with lower porosity into the membrane structure. The surface area and pore volume data are presented in Figure 3f,g, respectively. The trend in BET surface area shows the same pattern observed in the powder samples, demonstrating a decreasing trend as pore diameter increases. However, the surface area contributed by large pores (diameter > 20 nm) shows a gradual increase from MPC‐5 to MPC‐33, followed by a decline in MPC‐38. Furthermore, we carried out a detailed analysis of the pore volume of the membrane electrodes. To better understand the pore volume characteristics of the membrane electrodes, we conducted a detailed analysis using both N_2_ adsorption/desorption and mercury porosimetry. As the pore diameter increases, the pore volume associated with micropores (diameter < 2 nm) gradually decreases, dropping from 0.46 cm^3^ g^−1^ in MPC‐5 to 0.22 cm^3^ g^−1^ in MPC‐38. Conversely, the pore volume for mesopores initially increases from MPC‐5 to MPC‐33, before decreasing in MPC‐38. More specifically, the volume of the small mesopores (diameter 2–20 nm) increases, from 1.57 cm^3^ g^−1^ in MPC‐5 to 3.26 cm^3^ g^−1^ in MPC‐33, and then decreases to 2.67 cm^3^ g^−1^ in MPC‐38. The same trend is observed for large mesopores (diameter > 20 nm), although the changes are more pronounced. For example, the pore volume of MPC‐33 (2.68 cm^3^ g^−1^) is more than five times greater than that of MPC‐5 (0.41 cm^3^ g^−1^). Since the NIPS method was applied consistently under identical conditions following the preparation of carbon powders, the differences in macropore (> 200 nm) volume between samples were minimal, as shown in Figure 3g. Consequently, the total pore volume of the carbons generally increased, except for MPC‐38. These results indicate that for MPC‐33 and MPC‐38, not only is the total pore volume high, but a significant portion of this volume is derived from large mesopores. All the BET surface area and pore volume data of the membrane electrodes are summarized in **Table** **1**. These findings unequivocally suggest that these carbon membranes share similar macroporous structures, providing a consistent framework for the diffusion of oxygen and the deposition of Li_2_O_2_ during discharge. However, variations in the mesoporous structure of membrane electrodes could potentially lead to differences in electrolyte loading, capacity, and energy density.

**Table 1:** Surface area and pore volume of different membrane electrodes measured by N_2_ adsorption/desorption and Hg porosimetry.

Membrane electrode	BET surface area [m^2^ g^−1^]	Pore volume [cm^3^ g^−1^]				
		Micropore	Small mesopore	Large mesopore	Macropore	Total
MPC‐5	1118	0.46	1.57	0.41	3.29	5.73
MPC‐10	1070	0.4	2.63	1.05	2.9	6.98
MPC‐18	1035	0.37	3.11	1.58	3.42	8.48
MPC‐33	913	0.25	3.26	2.68	3.42	9.61
MPC‐38	595	0.22	2.67	2.18	2.87	7.94

### 2.6 Application of Free‐Standing Membrane Electrodes in LOBs

#### 2.6.1 Discharge Capacity Estimation of the Carbon Electrodes

**Figure 4:**
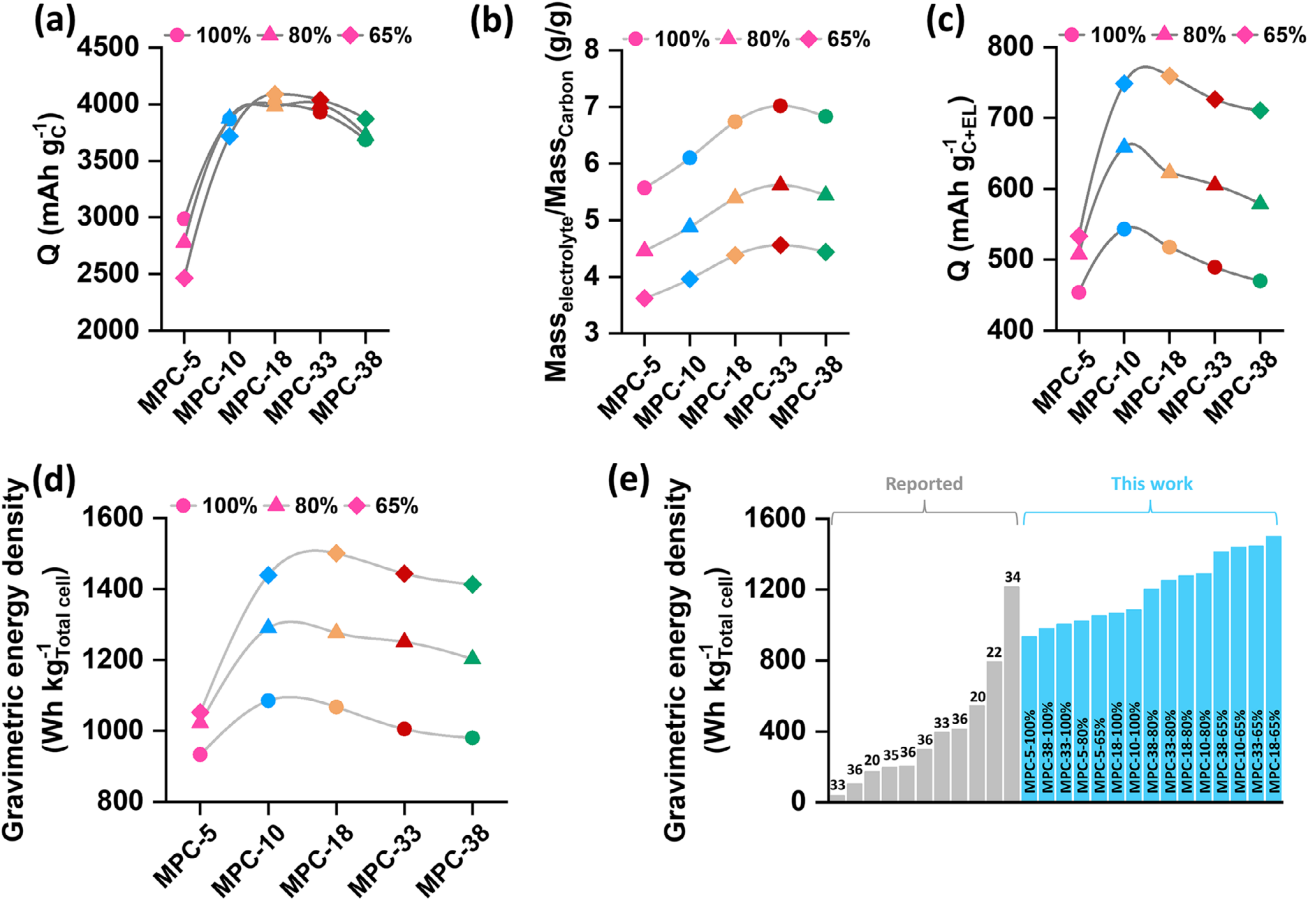
a) Specific capacity (normalized to carbon mass), b) Electrolyte to carbon mass ratio, c) Specific capacity (normalized to carbon + electrolyte mass), and d) Gravimetric energy density (normalized to total cell mass) of different electrodes with different electrolyte loading amounts. e) Comparison of the gravimetric energy density (normalized to total cell mass) reported in this work with a few selected earlier reports with high values.

The discharge performance of positive electrodes comprising various mesoporous carbon materials was systematically investigated using pouch‐type LOB cells under three controlled electrolyte loadings, corresponding to 100%, 80%, and 65% of the total pore volume of each electrode. Detailed cell configurations are provided in the Experimental methods, and the masses of the various cell components are shown in Table S2 (Supporting Information). For the cell tests, an electrolyte containing 0.5 m lithium bis(trifluoromethanesulfonyl)imide (LiTFSI), 0.5 m lithium nitrate (LiNO_3_), and 0.2 m lithium bromide (LiBr) dissolved in tetraethylene glycol dimethyl ether (TEGDME) was employed. TEGDME was chosen for its stability against both electrodes and its high boiling point, which prevents solvent loss in the open‐cell configuration of LOBs.^[^ ^28^ ^]^ Each salt served a distinct function. LiTFSI in the electrolyte ensures high ionic conductivity, LiNO_3_ stabilizes the Li anode and promotes solution‐phase discharge, suppressing carbon surface passivation, and LiBr reduces charge overpotential while further stabilizing the Li surface.^[^ ^29^, ^30^, ^31^, ^32^ ^]^ Galvanostatic discharge was performed at a current density of 0.4 mA cm^−2^ to a cut‐off voltage of 2.0 V versus Li/Li⁺. The discharge voltage profiles obtained under each electrolyte condition are presented in Figures S12–S14 (Supporting Information), while the resulting specific capacities, normalized to the carbon mass, are summarized in **Figure** **4**a. All samples, with the exception of MPC‐5 (pore volume < 6 cm^3^ g^−1^), delivered specific capacities exceeding 3500 mAh g^−1^. Figures S15–S17 (Supporting Information) further illustrate the correlation between discharge capacity and total pore volume. Interestingly, the results reveal that an increase in pore volume does not linearly translate to higher specific capacity. From MPC‐5 to MPC‐18, via MPC‐10, an increase in specific capacity is observed. However, beyond this range, as both pore diameter and pore volume further increase (MPC‐33), no additional capacity improvement is noted. Nevertheless, in the case of MPC‐38, the reduction in capacity can be attributed to lower pore volume compared to MPC‐18 and MPC‐33. This non‐monotonic trend suggests that excessively large pore volumes may lead to premature cell failure, primarily due to early passivation of the carbon surface by electronically insulating Li_2_O_2_, well before complete pore filling. Furthermore, larger pore volumes inherently necessitate increased electrolyte volumes to ensure adequate filling, which can offset the energy density gains achieved via higher capacities. Figure 4b and Table S3 (Supporting Information) present the electrolyte‐to‐carbon mass ratios corresponding to three electrolyte loading levels, 100%, 80%, and 65% of the total pore volume, of the respective carbon electrodes. As expected, an increase in electrode pore volume leads to a rise in electrolyte mass per unit mass of electrode. To assess the practical implications of this, the specific capacities of the LOB cells were recalculated by incorporating the actual electrolyte masses under each condition. With the exception of MPC‐5, which has the lowest pore volume, the inclusion of electrolyte mass in the specific capacity normalization yields minimal variation across other electrode types (Figure 4c; Table S3, Supporting Information). Notably, a declining trend in specific capacity is observed from MPC‐10 to MPC‐38 when normalized to the combined mass of carbon and electrolyte, indicating that pore volumes beyond an optimum limit may lead to diminishing returns in capacity enhancement.

This trend is also evident in the gravimetric energy density profiles shown in Figure 4d and detailed in Table S3 (Supporting Information). These results reveal that increasing electrode porosity does not inherently translate into higher gravimetric energy density at the cell level, where masses of all the cell components, including the electrolyte mass (Table S2, Supporting Information), were considered. On the contrary, the gravimetric energy density exhibits a more pronounced dependence on electrolyte mass. For instance, in the case of MPC‐18, the gravimetric energy density increases markedly from ≈1050 Wh kg^−1^ at 100% electrolyte‐loading to an unprecedented value of 1500 Wh kg^−1^ under 65% electrolyte‐loading. This emphasizes the critical role of electrolyte volume minimization in achieving high energy densities. Therefore, instead of continuously increasing pore volume, which enhances electrolyte demand, a more effective strategy for practical LOB design is to optimize electrode architecture in tandem with lean electrolyte conditions. Figure 4e and Figure S18 (Supporting Information) present a comparative analysis of the gravimetric energy densities achieved in this study against those reported for LOB cells in the literature.^[^ ^20^, ^22^, ^33^, ^34^, ^35^, ^36^ ^]^ The areal capacities and areal current densities of the cells mentioned in Figure 4e are shown in Table S4 (Supporting Information). Notably, the pore‐optimized carbon electrodes developed here deliver cell‐level gravimetric energy densities that surpass nearly all previously reported values to a large extent, with only one exception. This clear performance advantage underscores the effectiveness and novelty of the pore‐structure engineering strategy in advancing practical, high‐energy LOB systems.

#### 2.6.2 Discharge/Charge Cycling Test

**Figure 5:**
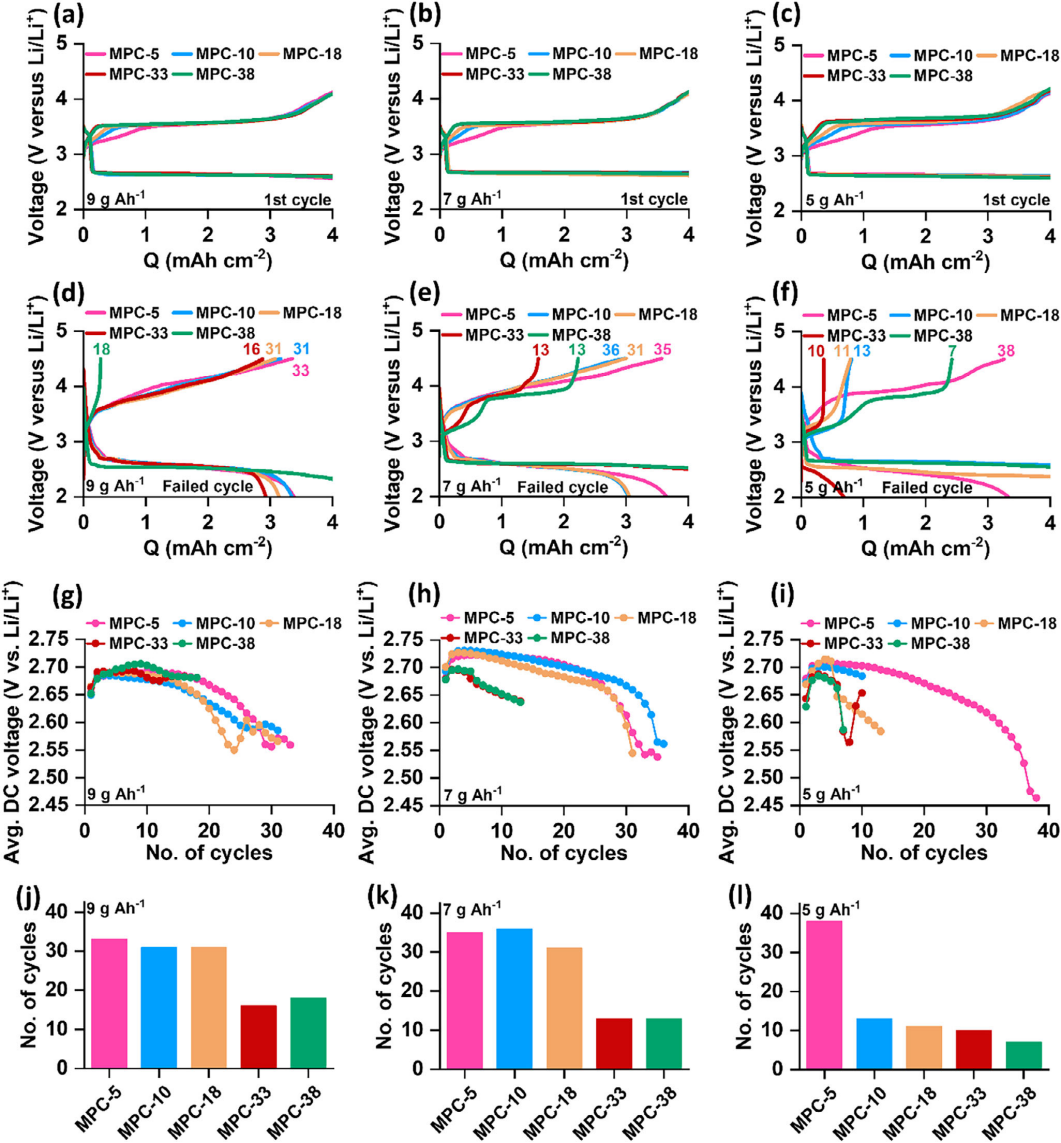
First cycle discharge/charge potential profiles of different carbon electrodes with a limited capacity of 4 mAh cm^−2^ under a) 9 g Ah^−1^, b) 7 g Ah^−1^, and c) 5 g Ah^−1^ electrolyte loadings. Discharge/charge potential profiles for the failed cycle of the same electrodes under d) 9 g Ah^−1^, e) 7 g Ah^−1^, and f) 5 g Ah^−1^ electrolyte loadings. Comparison of average discharge voltage over the cycles of different electrodes under g) 9 g Ah^−1^, h) 7 g Ah^−1^, and i) 5 g Ah^−1^ electrolyte loadings. j–l) Comparison of the cycle numbers of the electrodes with different electrolyte loadings.

Following the initial capacity assessment under different levels of electrolyte loading, the carbon electrodes underwent repeated discharge/charge cycling experiments. Pouch‐type LOBs were employed for these cycling tests, with cell capacities restricted to 4 mAh cm^−2^. To evaluate the impact of electrode pore volume and lean amount of electrolyte loading on the cycle life, the electrolyte mass was meticulously adjusted to three different levels: ≈5, ≈7, and ≈9 g Ah^−1^. **Figure** **5**a–f compares the galvanostatic voltage profiles of cells with different electrodes under varying levels of electrolyte loading for both the 1st cycle and the specific cycle at which either the cell stopped functioning due to reaching the cut‐off voltage or the discharge capacity dropped below 80% of the set capacity (depicted as cell death). In Figure 5a–c, it is evident that all carbon electrodes exhibited a consistent discharge voltage at ≈2.65 V versus Li/Li⁺ in the 1st cycle. Nevertheless, there were differences in charge voltage profiles of the carbons depending on the electrolyte loading.

When 9 g Ah^−1^ electrolyte was employed, throughout most of the charge, for all the electrodes, the voltage remained stable at 3.5–3.6 V versus Li/Li⁺, which is more evident from the magnified image in Figure S19 (Supporting Information). However, as the electrolyte amounts decreased, the charge voltage became higher for carbons with a larger pore diameter, as depicted in Figures S20 and S21 (Supporting Information). Specifically, the differences in the charge voltage among different electrodes are quite apparent in the case of 5 g Ah^−1^ in Figure S21 (Supporting Information). Figures S22–S26 (Supporting Information) further illustrate that the electrolyte loading amount has minimal effect on the discharge/charge potential in the cases of MPC‐5 and MPC‐10. However, for MPC‐18, MPC‐33, and MPC‐38, the charge voltage is higher for a lower amount of electrolyte. These results indicate that for carbons with larger pore diameter and pore volume, the resistance is higher during charging under a lean amount of electrolyte 5 g Ah^−1^. N_2_ adsorption/desorption analysis showed that the carbon electrodes with a larger pore size have a lower surface area. Consequently, the interfacial area between the electrode and electrolyte, specifically between Li_2_O_2_ and the electrolyte (the reaction site for Li_2_O_2_ decomposition), is also decreased. This reduction in interfacial area leads to an increase in effective current density. Moreover, as the amount of electrolyte decreases, the interfacial area further diminishes. This is why carbon electrodes with larger pore diameters and volumes exhibit higher charging voltages under lean electrolyte conditions.

More interesting results emerge regarding the failure modes of the cells depending on the electrolyte loading. Figure 5d shows the voltage profiles of all the cells in 9 g Ah^−1^ electrolyte‐loading for the specific cycle where the cell died. While cells with MPC‐5, MPC‐10, MPC‐18, and MPC‐33 showed a gradual increase in both discharge and charge voltage polarizations and capacity decay, the cell with MPC‐38 showed a sudden vertical spike in charge voltage, leading to cell failure. This sudden increase in the charge voltage can be attributed to the loss of electrolyte from the electrode pore and increased resistance in the cell. This observation becomes more obvious when the electrolyte loading amounts were decreased to 7 and 5 g Ah^−1^. In Figure 5e, it is observed that the cells with both the larger pore carbons, MPC‐33 and MPC‐38, failed due to a sudden increase in charge voltage. However, the cells with relatively smaller pore carbons showed a gradual decay in capacity. When the electrolyte loading was minimum at 5 g Ah^−1^, except for the cell with the smallest pore carbon MPC‐5, all other cells in Figure 5f showed a voltage spike during charge. Figure 5g–i presents the evolution of average discharge voltage over cycling for cells incorporating different carbon electrodes under electrolyte loadings of 9, 7, and 5 g Ah^−1^. At the highest electrolyte loading (9 g Ah^−1^), the average discharge voltages remain relatively consistent across all electrode types, indicating sufficient ionic conductivity and electrode filling. However, under leaner electrolyte conditions (7 and 5 g Ah^−1^), a marked decrease in average discharge voltage is observed for MPC‐33 and MPC‐38. Therefore, in the cases of the cells with carbons having a large pore diameter and pore volume, lean amounts of electrolyte impart higher voltage polarization and lead to sudden cell failure due to large resistance originating from the loss of electrolyte from the pores. The energy efficiency of the cells, defined as the ratio of discharge energy to charge energy, is presented in Figures S27–S29 (Supporting Information). A gradual decrease in energy efficiency is observed as the carbon pore diameter increases. Since the cycling was performed under a fixed capacity condition, these differences can be attributed to variations in the average discharge and charge voltages. In particular, the lower efficiency of the larger‐pore carbons arises from their lower average discharge potential and higher average charge potential. The apparent sudden increase in energy efficiency for cells with the larger pore carbons results from incomplete charging caused by voltage spikes. The cycling performance data of various carbon electrodes with different electrolyte loadings are shown in Figure 5j–l. When the electrolyte loading was maintained at 9 and 7 g Ah^−1^, a general decreasing trend in cycling stability was observed as the pore size of the carbon increased: MPC‐5 ≥ MPC‐10, ≈ MPC‐18 > MPC‐33 > MPC‐38. Nevertheless, the most substantial differences in cycling stability were observed under the lowest electrolyte loading of ≈5 g Ah^−1^. While MPC‐5 maintained stability for 38 cycles, MPC‐10, MPC‐18, MPC‐33, and MPC‐38 could only cycle for 13, 11, 10, and 7 cycles, respectively. Considering the importance of both reducing the electrolyte content and increasing the cyclable capacity for high energy density of the cells, cells were cycled between 2.0‐4.5 V versus Li/Li⁺ with 100%, 80%, and 65% electrolyte loadings without any capacity limit. As shown in Figures S30–S32 (Supporting Information), capacity fade became progressively worse as the electrolyte content decreased, particularly for electrodes with larger pores. In most cases, cell failure was triggered by sudden voltage spikes during charging. These observations indicate that electrolyte depletion‐induced polarization, rather than gradual degradation of the electrode or electrolyte, is the primary cause of failure in LOB cells under lean‐electrolyte conditions, and the situation becomes worse as the electrolyte displacement becomes higher at higher cyclable capacity.

### 2.7 Investigation of Cell Failure

#### 2.7.1 Impedance Analysis During Cycling

Enhanced voltage polarization and sudden voltage spikes observed during charging cycles signify a progressive increase in cell impedance, ultimately leading to premature cell failure. To elucidate the origins of this impedance rise under lean‐electrolyte conditions, electrochemical impedance spectroscopy (EIS) was conducted across different cycles. To minimize the influence of the Li electrode, a solid ceramic separator was used together with a stable 4 m lithium bis(fluorosulfonyl)imide in 1,2‐dimethoxyethane (DME) electrolyte on the Li side, which is known to sustain several hundred cycles with negligible degradation compared to the positive electrode. Under these conditions, the major changes observed in Ohmic, interphasial, and charge‐transfer resistances can be reasonably attributed to processes occurring at the positive electrode. The corresponding Nyquist plots and fitted equivalent circuits are provided in Figures S33–S48 (Supporting Information). Figures S49a–c and S50a–c (Supporting Information) show the evolution of Ohmic resistance (R_Oh_) and charge‐transfer resistance (R_CT_), respectively, measured at full charge. At an electrolyte loading of 9 g Ah^−1^, all cells exhibited a gradual increase in R_Oh_, except for MPC‐38, which showed a steep rise starting around the 11th cycle, which is consistent with the abrupt voltage behavior observed in Figure 5d. Under lower electrolyte loadings (7 and 5 g Ah^−1^), three cells with higher‐pore‐volume carbons (MPC‐18, MPC‐33, and MPC‐38) demonstrated a sudden increase in R_Oh_, each marking the onset of cell death. It is worth mentioning that it is the sudden increase in R_Oh_, rather than its absolute value, that correlates with the voltage spike in the respective cell. These results indicate that Ohmic resistance growth, driven by electrolyte depletion in high‐pore‐volume electrodes, is the dominant contributor to cell death under lean‐electrolyte regimes. By contrast, R_CT_ did not show a direct correlation with cycle life and instead fluctuated during cycling. This behavior is attributed to the dynamic deposition and partial decomposition of insulating side products (e.g., lithium carbonate (Li_2_CO_3_) and lithium carboxylates), which transiently alter interfacial charge transfer properties. While R_CT_ generally trends upward with cycle number, its variability suggests a more complex interfacial degradation mechanism, distinct from the bulk electrolyte‐mediated failure reflected by R_Oh_.

#### 2.7.2 Online Electrochemical Mass Spectrometry for Gas Analysis

Subsequently, we focused on investigating cell degradation under different conditions. In our recent analytical investigation, we have indeed demonstrated enhanced parasitic reactions in LOBs under lean electrolyte conditions. The core principle of LOBs involves the reversible electrochemical reduction and evolution of O_2_ during discharge and charge cycles, respectively. As such, the assessment of O_2_ evolution serves as a standard measure to evaluate the reversibility of LOBs. However, alongside the desired O_2_ evolution, the occurrence of parasitic side reactions from both the electrode and electrolyte often results in the release of carbon dioxide (CO_2_) gas in LOB systems. Therefore, quantifying the evolved gases offers a comprehensive insight into the reactions within the positive electrode and their impact on the cycling stability of the cells.

**Figure 6:**
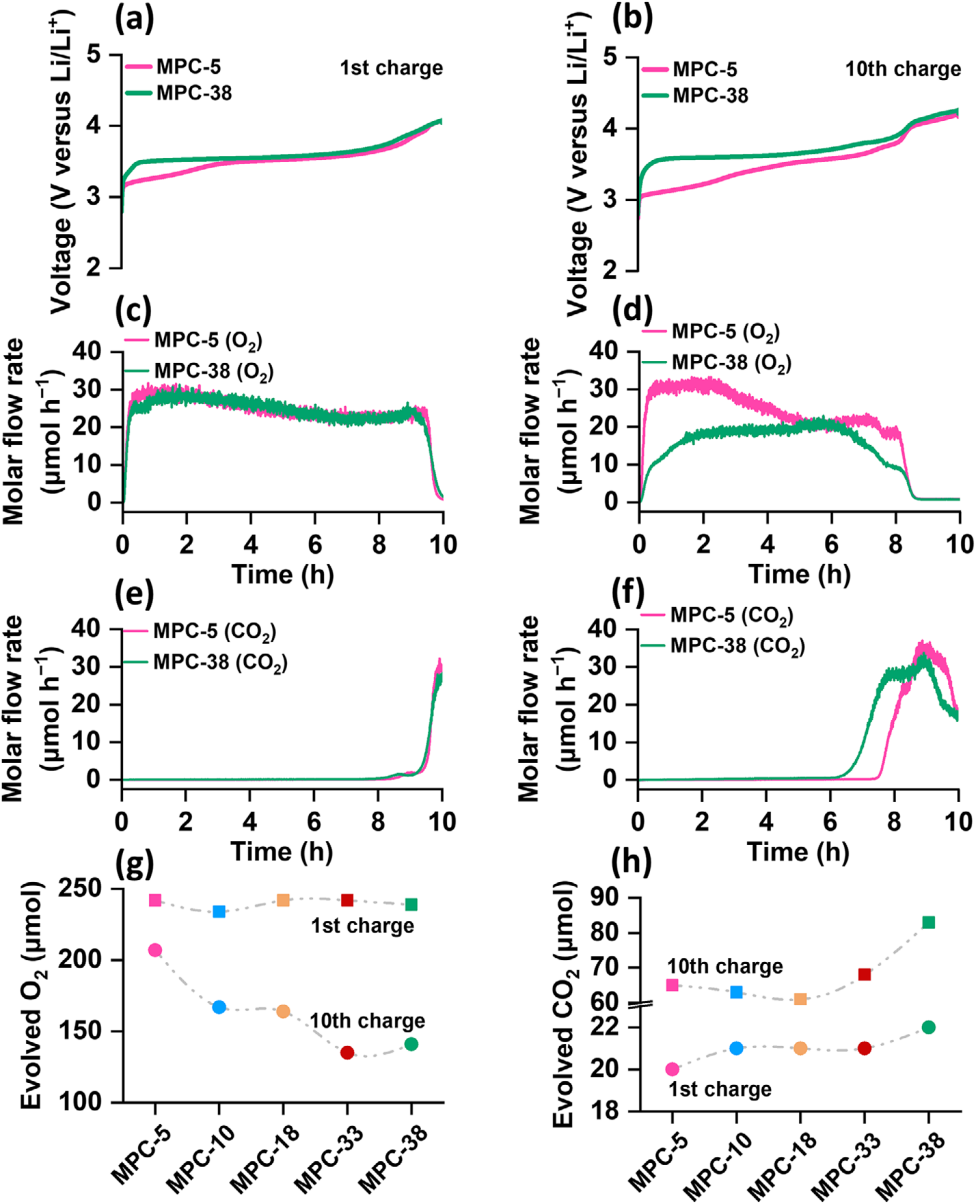
Charging voltage profiles a,b), and the corresponding O_2_ c,d) and CO_2_ e,f) evolution rates of MPC‐5 and MPC‐10 for the first and tenth cycles, respectively. Comparison of the quantities of evolved g) O_2_ and h) CO_2_ for different electrodes in the first and 10^th^ charge.

To quantify the evolved gases, we conducted an online electrochemical mass spectrometry (online MS) analysis. For the online MS experiments, we utilized a specialized two‐compartment cell design, wherein the Li negative electrode is isolated from the positive electrode using a glass ceramic separator. Figure S51 (Supporting Information) shows a schematic design of the flow‐type cell used for this MS analysis. This cell design ensures the exclusive estimation of evolved gases from the positive electrode. Figures S52–S61 (Supporting Information) present the voltage profiles of LOBs using different electrodes, along with the corresponding rates of gas evolution for the 1st and 10th cycles. The outcomes depicted in **Figure** **6**a,b compare the voltage profiles, and Figure 6c–f compare the gas evolution rates of the carbons with the smallest and largest pore diameter, MPC‐5 and MPC‐38, as examples, for the 1st and 10th charge processes, respectively. Figure 6c–e indicate that during the 1st charge, there were no significant differences in the rates of O_2_ and CO_2_ evolution between MPC‐5 and MPC‐38. However, at the 10th charge, a substantial decrease in O_2_ evolution rate is observed in Figure 6d and f for MPC‐38 compared to MPC‐5. Additionally, the charge voltage for the cell with MPC‐38 in the 10th charge (see Figure 6b) is higher than that with MPC‐5, potentially triggering enhanced decomposition of both the electrolyte and the electrode. Indeed, the cell with MPC‐38 exhibited a much earlier onset of CO_2_ evolution at ≈60% state of charge (SOC), compared to 75% SOC in the case of MPC‐5. Figure 6e and f illustrate the quantitative estimation of the total amounts of O_2_ and CO_2_ evolved during the 1st and 10th cycles for all samples. The results in Figure 6g indicate that during the 1st charge, all cells exhibited similar amounts of O_2_ evolution, with an estimated yield of 80±2% O_2_ (see Figure S62, Supporting Information). However, notable differences in O_2_ evolution profiles emerged during the 10th charge. A gradual decrease in O_2_ yield was observed as the pore diameter of the carbon increased. For instance, the LOB with MPC‐5 displayed an O_2_ evolution yield of 69% during the 10th charge, decreasing to 56±1% for MPC‐10 and MPC‐18, and 46±1% for MPC‐33 and MPC‐38. Consistent with the O_2_ evolution profiles, all cells exhibited similar CO_2_ evolution during the 1st charge, as shown in Figure 6h. However, a significant increase in the quantity of evolved CO_2_ during the 10th charge indicated enhanced parasitic reactions, particularly pronounced for electrodes with larger pore carbons, MPC‐33 and MPC‐38. This gas analysis provides compelling evidence of the superior O_2_ reversibility and increased resistance to side reactions in positive electrode materials with relatively smaller pore diameters under lean electrolyte conditions. Depletion of electrolytes from the electrodes with larger pore carbons under these conditions, which worsens during cycling, leads to increased R_Oh_ and voltage polarization, triggering enhanced electrolyte decomposition.

#### 2.7.3 Analysis of the Carbon Electrode Pore Structure During Cycling

The N_2_ adsorption/desorption isotherms and BJH pore size distribution curves for all the carbon electrodes at various cycle states are shown in Figures S63–S67 (Supporting Information). Figure S68a,b (Supporting Information) compare the BET surface area and pore volume, respectively, of all the electrodes in their pristine state and after selected cycles. The results indicate that, except for MPC‐33 and MPC‐38, there is a gradual decrease in both BET surface area and pore volume during cycling for the other carbon electrodes. During prolonged cycling of LOB cells, electrolyte degradation leads to the formation and accumulation of solid lithium‐containing byproducts that progressively obstruct the porous structure of the electrode. This effect is particularly exacerbated in carbon electrodes with smaller pore diameters and limited pore volumes (e.g., MPC‐5, MPC‐10, MPC‐18), where the confined pore space is more easily blocked. The higher surface area of these smaller‐pore carbons exposed to the oxidizing environments increases their susceptibility to parasitic reactions, further accelerating electrode passivation. Moreover, due to more effective electrolyte filling, smaller‐pore electrodes show relatively lower R_Oh_ and reduced charge voltages, which are insufficient to decompose accumulated side products, as reflected by minimal CO_2_ evolution. This results in progressive pore blockage over repeated cycles. Conversely, larger‐pore electrodes such as MPC‐33 and MPC‐38 exhibit higher charge voltages, which facilitate partial oxidative decomposition of the parasitic species, thereby preserving porosity to a greater extent. Nonetheless, the retention of open pore structure in these electrodes does not signify improved reversibility or long‐term stability, but rather indicates a continuous and more aggressive interfacial degradation.

## 3 Discussions

The findings presented above highlight a critical trade‐off between electrode porosity, gravimetric energy density, and cycling stability in LOBs. Electrodes with low pore volume, such as MPC‐5, consistently exhibit low gravimetric energy density, irrespective of the electrolyte‐loading level, due to limited space for Li_2_O_2_ deposition. As the pore volume increases to intermediate levels (MPC‐10 and MPC‐18), the gravimetric energy density improves significantly. However, further increases in pore volume (MPC‐33 and MPC‐38) result in a decline in gravimetric energy density. This reduction is attributed to the greater electrolyte mass required to saturate the expanded pore network, which offsets the capacity gain and lowers the gravimetric energy density. This trend is consistent across varying electrolyte‐loading levels. Although decreasing the electrolyte volume improves gravimetric energy density across all samples, MPC‐10 and MPC‐18 still maintain higher values. Besides, the highly porous electrodes (MPC‐33 and MPC‐38) suffer from electrolyte depletion, high impedance growth, increased parasitic reactions, and shortened cycle life under lean electrolyte conditions. Overall, MPC‐10 and MPC‐18 represent an optimal balance between porosity and electrolyte utilization, resulting in superior gravimetric energy density and cycling stability. At 80% pore filling, they deliver gravimetric energy densities exceeding 1200 Wh kg^−1^, and sustain stable cycling for over 30 cycles under the capacity limiting condition of 4 mAh cm^−2^. These results suggest that tuning the pore volume and precisely controlling electrolyte loading are key design parameters for practical LOB electrodes. This interplay is qualitatively illustrated in Figure S69 (Supporting Information).

## 4 Conclusion

Our study underscores the critical importance of optimizing carbon porosity to achieve high capacity while balancing the electrolyte amount necessary for adequate electrode filling, thereby ensuring stable cycling of LOBs with high gravimetric energy densities. Through simulation and experimental validation, we found that although increasing electrode porosity correlates with higher absolute capacity, it does not guarantee high specific capacity or gravimetric energy density due to the significant electrolyte volumes required to saturate the pores. Besides, the cycling stability tests under three different lean electrolyte conditions revealed a declining trend in stability with reduced electrolyte content and increased carbon pore volume. As pore diameter and volume increased, cell failure occurred due to insufficient electrode filling and electrolyte depletion. EIS measurements during cycling showed that this depletion caused increased cell impedance, voltage polarization, and electrolyte degradation. Conversely, for carbons with very small pores and low pore volume, the issue of electrolyte depletion is less severe, but pore blockage and decreased porosity during cycling pose significant challenges. Therefore, our findings emphasize the need to optimize carbon electrode porosity to balance energy density and cycle life in LOBs. Subsequently, a pore‐optimized carbon electrode showed a very high gravimetric energy density exceeding 1500 Wh kg^−1^ and stable cycling under capacity‐limiting conditions. These insights offer substantial promise for advancing the development of carbon materials for the next generation of practical LOBs, providing enhanced performance and energy density.

## 5 Experimental Section

### Synthesis of Carbons

Mesoporous carbon structures were synthesized using a hard‐template approach, where magnesium oxide (MgO) particles of various sizes acted as the template, and the details of the synthesis procedure can be found elsewhere.^[^ ^37^, ^38^, ^39^ ^]^ MgO nanoparticles were synthesized, and the particle size was controlled by pyrolyzing magnesium acetate (Mg(CH_3_COO)_2_) and magnesium citrate (Mg_3_(C_7_H_7_O_7_)_2_) for different durations. Phenol resin was employed as the carbon precursor. The MgO and phenol resin mixture was thermally treated at 900 °C in a nitrogen atmosphere to form a carbon–MgO composite. The MgO component was subsequently removed by acid treatment with 1 m sulfuric acid, resulting in the formation of mesoporous carbons. The removal of MgO was confirmed by XRF (X‐ray Fluorescence) analysis, showing that the residual amount of MgO was 100 ppm or less. This carbon material underwent a final high‐temperature treatment at 1800 °C under nitrogen to improve its structural integrity.

### Fabrication of Self‐Standing Carbon Membrane Electrode

A self‐standing carbon gel‐based membrane was developed by utilizing carbon powder samples. The fabrication process involved the following steps:

### Fabrication of Self‐Standing Carbon Membrane Electrode–Slurry Preparation

The process starts with preparing a slurry consisting of 75 wt.% carbon powder, 5 wt.% single‐walled carbon nanotube (OCSiAl, TUBALL, CNT, average fiber diameter 1.6 nm, average length 5 µm), 5 wt.% carbon fiber (Nippon Polymer Sangyo Co., Ltd., CF, average fiber diameter 6 µm, average length 3 mm), 15 wt.% PAN, and NMP as a solvent to ensure uniform dispersion.

### Fabrication of Self‐Standing Carbon Membrane Electrode–Film Formation

The prepared slurry was then uniformly spread onto a sheet using a doctor blade technique. This method helps maintain a consistent film thickness across the entire surface.

### Fabrication of Self‐Standing Carbon Membrane Electrode–Pore Generation

Afterward, the sample underwent immersion in methanol (a poor solvent) and was transformed into a porous film through the nonsolvent‐induced phase separation (NIPS) method.

### Fabrication of Self‐Standing Carbon Membrane Electrode–Drying and Stabilization of the Film

The resulting film underwent solvent removal by drying at 80 °C for 10 h, followed by an infusibilization treatment at 230 °C for 3 h in an air‐circulating atmosphere using an oven DN411 (Yamato Scientific Co., Ltd.).

### Fabrication of Self‐Standing Carbon Membrane Electrode–Carbonization

Carbonization was the final step that was conducted in a box‐type furnace (Denken High Dental Co., Ltd.) under nitrogen flow (800 mL min^−1^) by gradually increasing the temperature to 1050 °C at a rate of 10 °C min^−1^, maintaining it at 1050 °C for 3 h, and then allowing the sample to cool to room temperature.

### Characterization of Self‐Standing Carbon Membrane Electrode

The pore structures of the samples underwent characterization using nitrogen adsorption/desorption (3 FLEX, Micromeritics Instrument Corp.), while macropore size distributions were determined via mercury porosimetry (AutoporeIV 9505, Shimadzu Co.). X‐ray diffraction (XRD) patterns of the carbon powders were obtained using an X‐ray diffractometer (SmartLab, Rigaku). Morphological analysis of the carbon powders was conducted using a Field‐emission scanning electron microscope (FE‐SEM, S‐4800, Hitachi). A laser Raman microscope (RamanTouch‐VIS‐NIR, Nanophoton) was employed to assess the graphitization of the carbons. Surface chemical characterization of the carbon samples was carried out using X‐ray photoelectron spectroscopy (ULVAC‐PHI, VersaProbe II). The transmission electron micrographs (TEM) were obtained from a Jeol JEM‐ARM200F.

### Lithium–Oxygen Cell Assembly and Discharge Performance Test

An electrolyte comprising of 0.5 m lithium bis(trifluoromethanesulfonyl)imide (LiTFSI; Kishida Chemical Co., Ltd., purity >99.9%), 0.5 m lithium nitrate (LiNO_3_; Sigma–Aldrich Co., LLC, purity 99.99% trace metals basis), and 0.2 m lithium bromide (LiBr; Sigma–Aldrich Co., LLC, purity 99.995% trace metals basis) dissolved in tetraethylene glycol dimethyl ether (TEGDME; Kishida Chemical Co., Ltd., purity >99%) was utilized for cell tests. Both LiNO_3_ and LiBr salts were dried under vacuum at 120 °C for over 3 days before electrolyte preparation to ensure dryness. The water content of the electrolyte was verified to be less than 30 ppm through Karl Fischer titration. Self‐standing carbon membranes were utilized as the positive electrodes after undergoing vacuum drying at 100 °C for 12 h. Pouch‐type lithium–oxygen cells (2 × 2 cm^2^) were assembled in a dry room with a water content of less than 10 ppm. The cell assembly process involved stacking metallic lithium foil (Honjo Metal Co., Ltd.), a polyolefin‐based separator, a carbon electrode, and a gas diffusion layer (TGP‐H‐060, Toray, Japan) sequentially. Electrolyte injection into carbon electrodes was accomplished using the vacuum impregnation method, maintaining 3 different levels of electrolyte loading: ≈65%, 80%, and 100% of the pore volumes of the electrodes. A pressure of 100 kPa was applied to the cell by a spring coil. The lithium–oxygen cells were kept within an oxygen‐filled box, with oxygen gas continuously flowing at a rate of 80 mL min^−1^. Electrochemical discharge experiments were conducted using a battery test equipment (SD8, Hokuto Denko Corp.). The cutoff voltage was set at 2.0 V versus Li/Li⁺ at a current density of 0.4 mA cm^−2^.

### Lithium–Oxygen Cell Assembly and Discharge/Charge Cycling Test

For the cycling tests, the same ternary salt electrolyte was utilized. Lithium–oxygen cells were assembled inside a dry room where the water content was maintained below 10 ppm. The cell assembly comprised stacking a lithium–metal foil (2 × 2 cm^2^, thickness of 0.1 mm; Honjo Metal Co., Ltd.), a polyolefin‐based separator (2.2 × 2.2 cm^2^, thickness of 0.02 mm), a porous carbon electrode (2 × 2 cm^2^), and a gas‐diffusion layer. The gas‐diffusion layer used in the cell consisted of an array of carbon fibers having ≈10 µm diameter (thickness of 110 µm, TGP‐H‐030, Toray). Moreover, an array of Ni/Cu‐coated PET fibers ≈30 µm in diameter (thickness of 45 µm, Sui‐40‐9027, SEIREN Electronics Materials) served as the current collector. A ceramic‐based solid‐state separator (LICGC, thickness of 0.50 mm; Ohara, Inc.) was used to protect the lithium–metal negative electrode. This ceramic‐based solid‐state separator was placed between polyolefin‐based separators, and the same electrolyte was used for both the positive and negative electrode sides. Electrolyte injection into carbon electrodes was accomplished using the vacuum impregnation method, maintaining 3 different levels of electrolyte loading: ≈5, 7, and 9 g Ah^−1^. A pressure of 100 kPa was applied to the cell by a spring coil. Electrochemical experiments were conducted using a battery test equipment (TOSCAT, Toyo System Co., Ltd.). The limiting capacity and cutoff voltage were set at 4.0 mAh cm^−2^ and 2.0 V/4.5 V versus Li/Li⁺, respectively, with a current density of 0.4 mA cm^−2^. The lithium–oxygen cells were kept inside an oxygen‐filled box, with oxygen gas continuously flowing at a rate of 80 mL min^−1^.

### Online Mass Spectroscopic (MS) Analysis

High‐resolution mass spectrometry (MS) analysis was conducted using an MS instrument (M‐401GA, CANON ANELVA Corp.) configured in an online setup. An electrochemical flow cell, specifically designed for this purpose and with an inner volume of ≈24 mL (diameter = 70 mm; depth = 15 mm), was utilized for the analysis. This cell incorporated the same components as those detailed for the lithium‐oxygen cells mentioned earlier. The negative electrode was separated from the positive electrode with a glass ceramic separator. While the same ternary‐salt electrolyte was used for the positive electrode side, a 4 m lithium bis(fluorosulfonyl)imide (LiFSI) in dimethoxyethane (DME) electrolyte was used for the negative electrode. Prior to measurement, the cells were filled with oxygen (O_2_) gas and subjected to a discharge process until reaching a discharge capacity of 4 mAh cm^−2^. Subsequently, the test cell was purged with excess helium (He) gas at a flow rate of 50 mL min^−1^ for 1 min to remove any remaining O_2_. During the charging process, gas evolution was continuously monitored using He as the carrier gas at a flow rate of 5 mL min^−1^. For online MS analysis, the generated gases were directly transferred to the MS detector through a capillary tube (internal diameter: 0.05 mm, length: 7 m).

The reproducibility of the results was verified by performing all experiments in at least two independent runs.

## Conflict of Interest

The authors declare no conflict of interest.

## Supporting information



Supporting Information

## Data Availability

The data that support the findings of this study are available from the corresponding author upon reasonable request.
