# Supplementary material for: Quantitative Porosity Engineering of Carbon Electrode in Lithium–Oxygen Batteries with Cell‐Level Gravimetric Energy Density Over 1500 Wh kg−1
Source: Adv Sci (Weinh). 2025 Oct 15;13(1):e14406. doi: 10.1002/advs.202514406 (PMC12766985; doi:10.1002/advs.202514406)
Supplement: Supplementary file 1 — Supporting Information [file ADVS-13-e14406-s001.pdf]

Supporting Information

Quantitative Porosity Engineering of Carbon Electrode in Lithium–Oxygen Batteries with Cell-level Gravimetric Energy Density over 1500 Wh kg^{-1}

Arghya Dutta, Takashi Kameda, Junji Takada, Yuuka Nakajima, Takahiro Morishita, and Shoichi Matsuda**

Note S1.

Simulation of cell-level gravimetric energy density depending on the electrode pore volume, porosity, thickness, mass-loading, and electrolyte content:

A parametric simulation was performed to evaluate how electrode pore volume, mass loading, porosity, thickness, and electrolyte filling fraction influence the achievable gravimetric energy density of LOB cells. The model was constructed using experimentally relevant constants: Li_2O_2 density ($\rho_{Li_2O_2} = 2.31\ g\ cm^{-3}$), electrolyte density ($\rho_{El} = 1.1\ g\ cm^{-3}$), theoretical specific capacity of Li_2O_2 ($Q_{th} = 1168.4\ mAh\ g^{-1}$), and an average discharge potential ($V_{avg} = 2.7\ V$). A fixed areal mass of other components ($M_{Other} = 7.74\ mg\ cm^{-2}$), obtained from real cells, was included to represent the masses of gas diffusion layer, separator, Li electrode, current collector etc. As a reference, the bulk density of the carbon electrode is considered to be 0.2 g cm^{-3}.

The variable parameters are the pore volume (v_p; $cm^3\ g^{-1}$), areal mass loading (M_C; g cm^{-2}), fraction of pore volume filled by the electrolyte (f_{El}), electrode thickness (*t*; cm), and porosity (ε) of the electrode.

Li_2O_2 mass per area (g cm^{-2}) formed if pores are fully filled by the product:

$$M_{Li_2O_{2(Area)}} = \rho_{Li_2O_2} \times v_p \times M_C$$

Areal capacity (mAh cm^{-2}):

$$Q_{Area} = M_{Li_2O_{2(Area)}} \times Q_{th}$$

Areal energy (mWh cm^{-2}):

$$E_{Area} = Q_{Area} \times V_{avg}$$

Electrolyte mass per area (for filling fraction f_{El}) (g cm^{-2}):

$$M_{El_{(Area)}} = f_{El} \times \rho_{El} \times v_p \times M_C$$

Total cell mass per area (with electrolyte) (g cm^{-2}):

$$M_{Total} = M_C + M_{Other} + M_{El_{(Area)}}$$

Gravimetric energy density (mWh g^{-1}):

Using the areal quantities above

$$\textbf{Gravimetric energy density} = \frac{E_{Area}}{M_{Total}} = \frac{\rho_{Li_2O_2} \times v_p \times M_C \times Q_{th} \times V_{avg}}{M_C + M_{Other} + (f_{El} \times \rho_{El} \times v_p \times M_C)}$$

Contour plots:

1. Contour of gravimetric energy density against M_C and v_p:

For this plot, both M_C and v_p are varied, while f_{El} is kept at 100%.

2. Contour of gravimetric energy density against f_{El} and v_p:

For this plot, both f_{El} and v_p are varied, while M_C is kept at 4 mg cm^{-2}.

3. Contour of gravimetric energy density against *t* and porosity *ε*:

In this case, $M_C = 0.2\ (1 - \varepsilon)$ and $M_{Li_2O_{2(Area)}} = \rho_{Li_2O_2}\ \times\ \varepsilon\ \times t$

For this plot, both *t* and *ε* are varied, while f_{El} is kept at 100%.

4. Contour of gravimetric energy density against f_{El} and *ε*:

For this plot, both f_{El} and *ε* are varied, while *t* is kept at 200 μm.

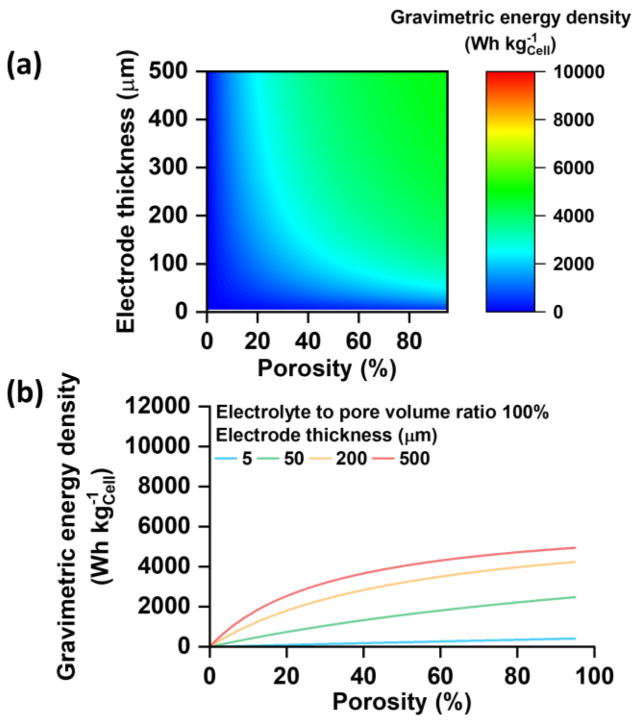

Figure S1. (a) Contour map of the simulated gravimetric energy density (total cell mass) against porosity and electrode thickness, with 100% of the pores filled with electrolyte. Electrode mass-loading for a 100 μm-thick electrode is considered to be 2 mg cm^{-2}. (b) Line plots of simulated gravimetric energy density against porosity for four specific values of electrode thickness.

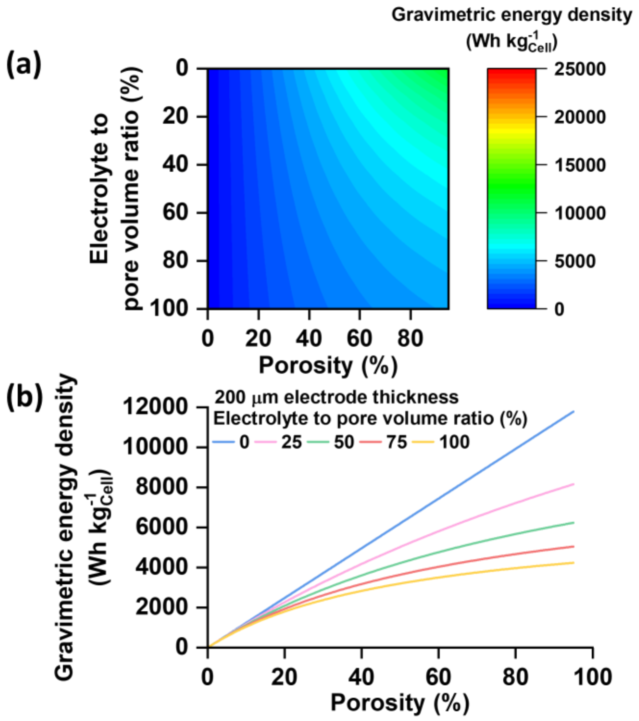

Figure S2. (a) Contour map of the simulated gravimetric energy density (total cell mass) against porosity and electrolyte loading amounts for an electrode of 200 μm thickness. Electrode mass-loading for the 200 μm-thick electrode is considered to be 4 mg cm^{-2}. (b) Line plots of simulated gravimetric energy density against porosity for five specific values of electrolyte loading.

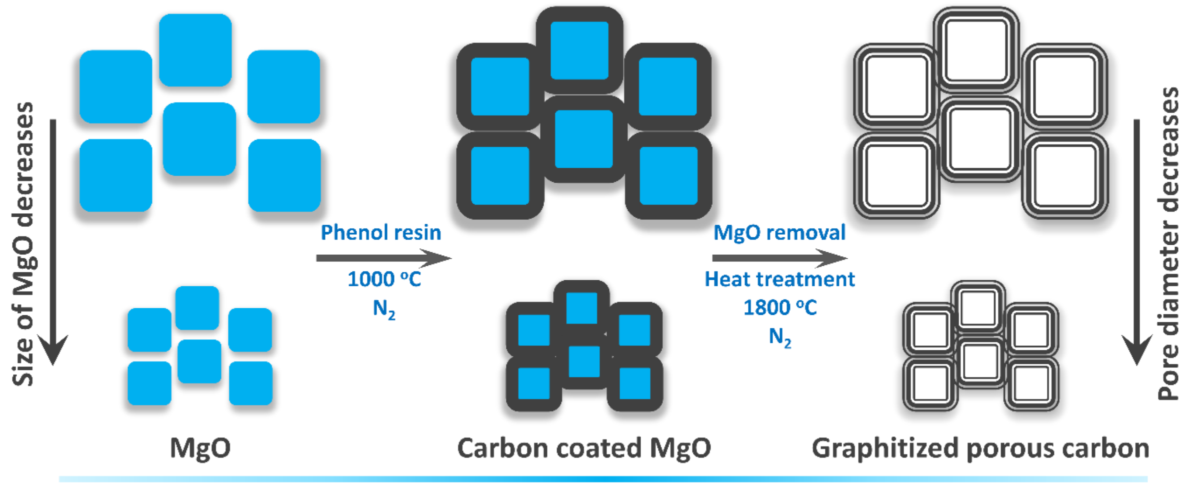

Figure S3. Schematic illustration of the hard-templated synthesis of the porous carbon powders.

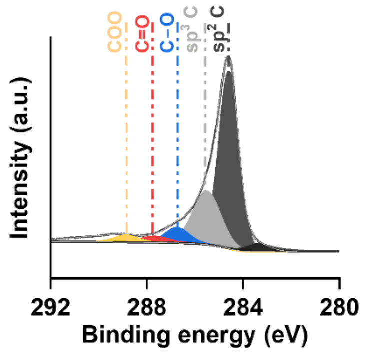

Figure S4. C1s XPS spectrum of MPC-10 as a representative sample.

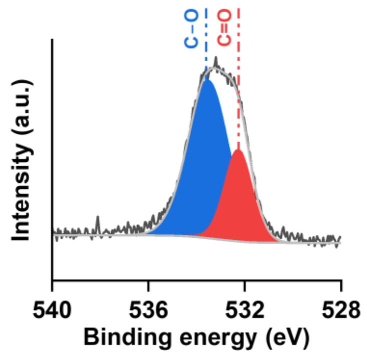

Figure S5. O1s XPS spectrum of MPC-10 as a representative sample.

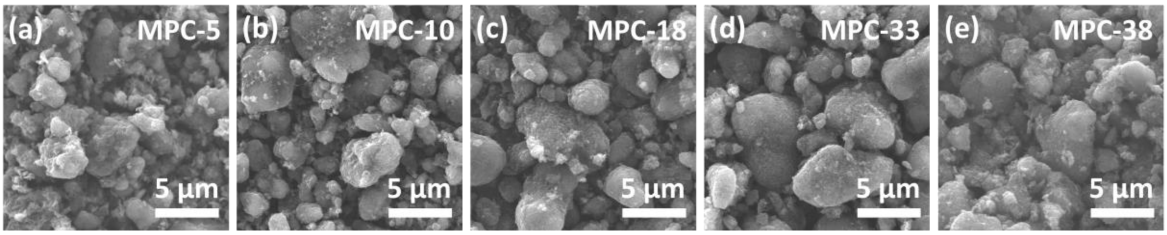

Figure S6. Low magnification SEM images of different porous carbon powders.

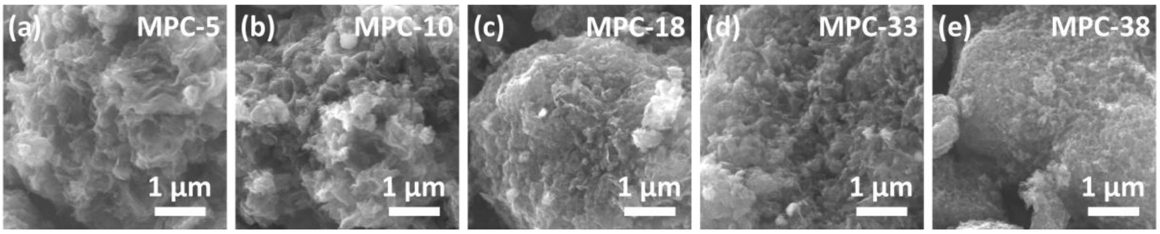

Figure S7. High magnification SEM images of different porous carbon powders.

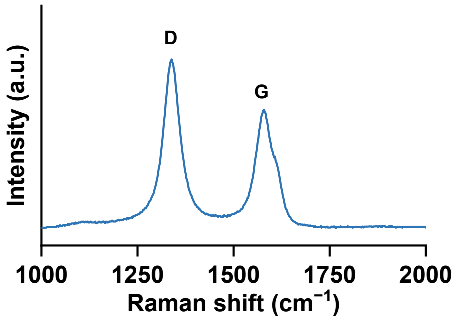

Figure S8. Raman spectrum of MPC-10 as a representative sample.

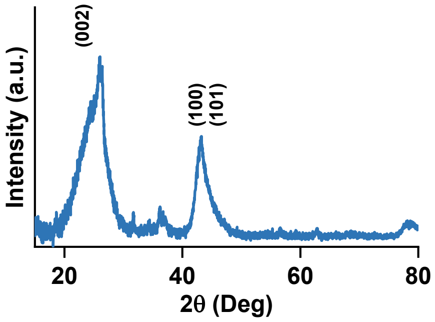

Figure S9. XRD pattern of MPC-10 as a representative sample.

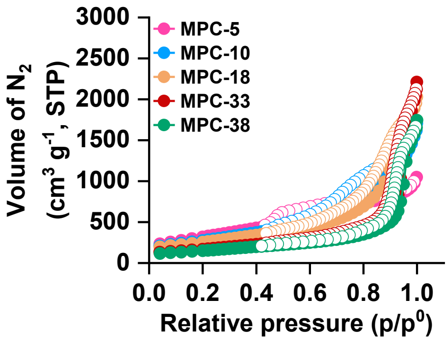

Figure S10. N_2 adsorption/desorption isotherms of different carbon membranes.

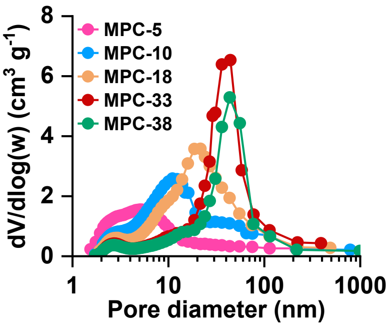

Figure S11. BJH pore size distribution (from adsorption data) of different carbon membranes.

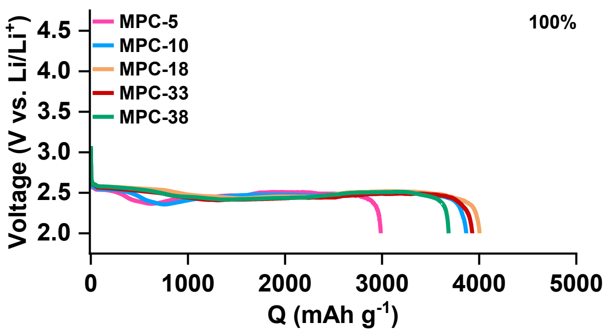

Figure S12. Discharge voltage profiles of different carbon electrodes with 100% electrolyte filling of the pores.

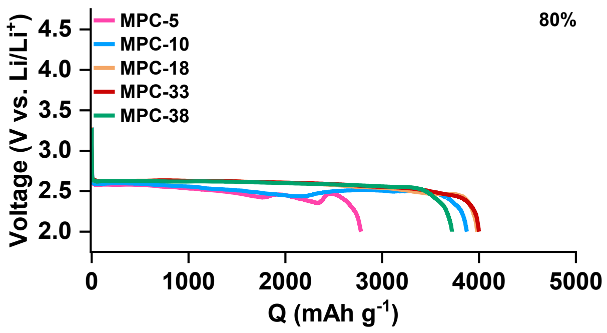

Figure S13. Discharge voltage profiles of different carbon electrodes with 80% electrolyte filling of the pores.

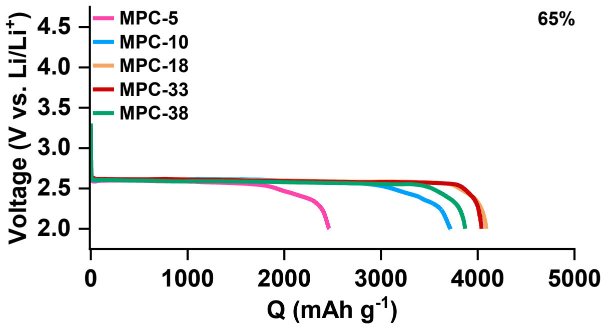

Figure S14. Discharge voltage profiles of different carbon electrodes with 65% electrolyte filling of the pores.

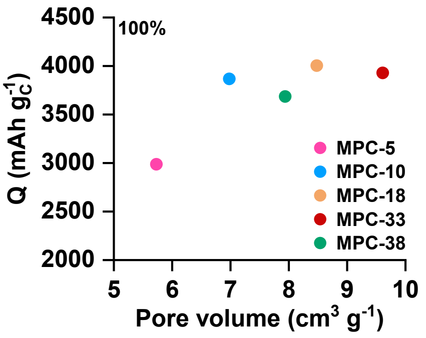

Figure S15. Comparison of specific capacities (normalized to carbon mass) of different carbon electrodes with 100% electrolyte filling of the pores.

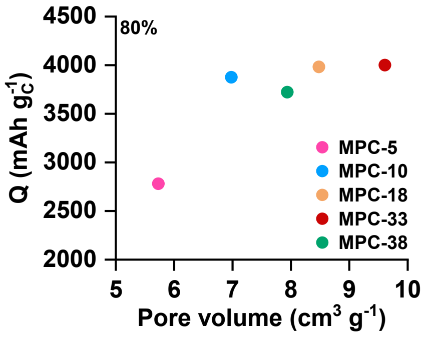

Figure S16. Comparison of specific capacities (normalized to carbon mass) of different carbon electrodes with 80% electrolyte filling of the pores.

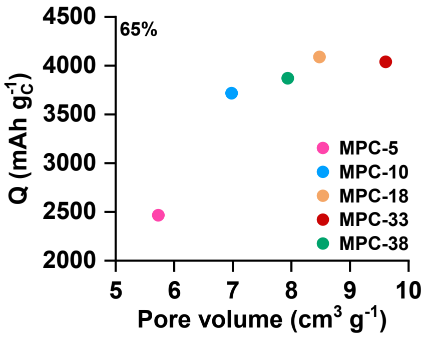

Figure S17. Comparison of specific capacities (normalized to carbon mass) of different carbon electrodes with 65% electrolyte filling of the pores.

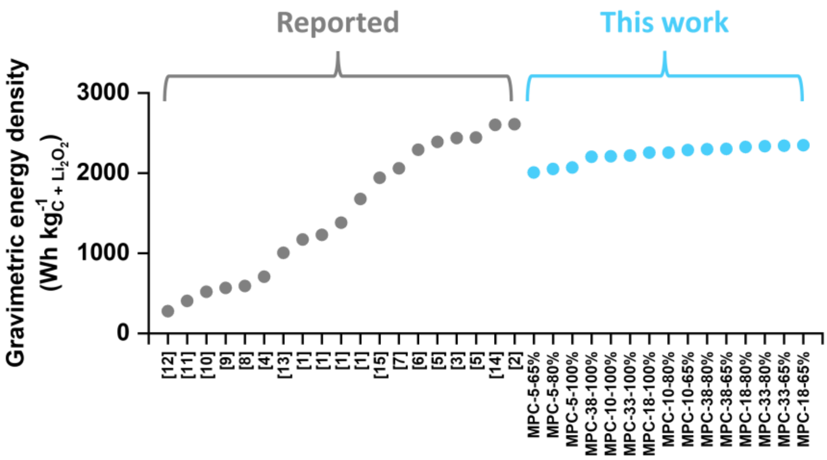

Figure S18. Comparison of gravimetric energy density (normalized to carbon + Li_2O_2 mass) reported in this work with a few selected earlier reports with high values. The mass of the electrolyte is not included in this calculation.[1–15]

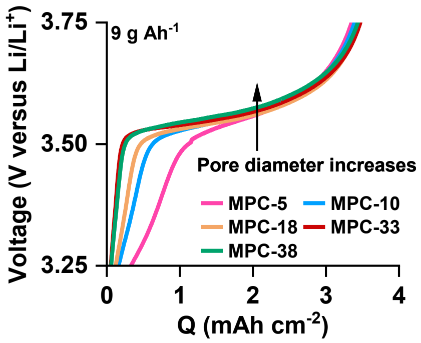

Figure S19. Magnified view of the first cycle charging voltage profiles of different electrodes cycled with a fixed capacity of 4 mAh cm^{-2} with an electrolyte loading amount of 9 g Ah^{-1}.

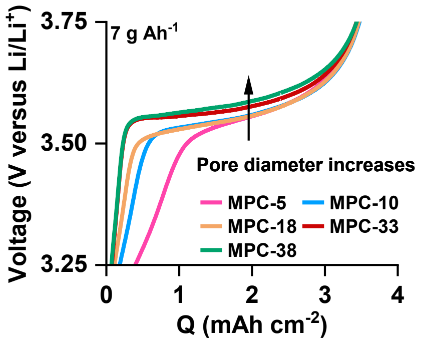

Figure S20. Magnified view of the first cycle charging voltage profiles of different electrodes cycled with a fixed capacity of 4 mAh cm^{-2} with an electrolyte loading amount of 7 g Ah^{-1}.

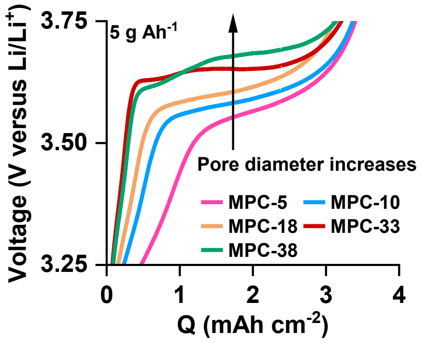

Figure S21. Magnified view of the first cycle charging voltage profiles of different electrodes cycled with a fixed capacity of 4 mAh cm^{-2} with an electrolyte loading amount of 5 g Ah^{-1}.

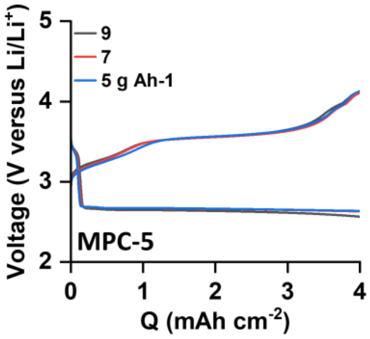

Figure S22. First cycle voltage profiles of MPC-5 electrode cycled with a fixed capacity of 4 mAh cm^{-2} and different electrolyte loading amounts.

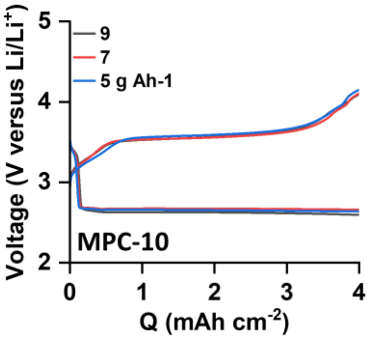

Figure S23. First cycle voltage profiles of MPC-10 electrode cycled with a fixed capacity of 4 mAh cm^{-2} and different electrolyte loading amounts.

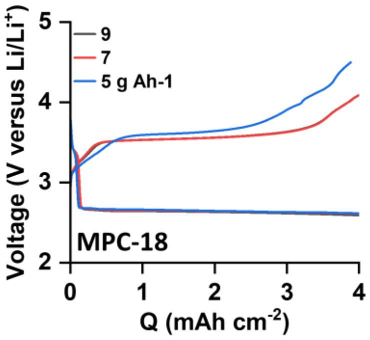

Figure S24. First cycle voltage profiles of MPC-18 electrode cycled with a fixed capacity of 4 mAh cm^{-2} and different electrolyte loading amounts.

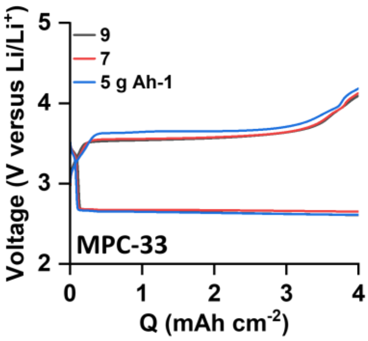

Figure S25. First cycle voltage profiles of MPC-33 electrode cycled with a fixed capacity of 4 mAh cm^{-2} and different electrolyte loading amounts.

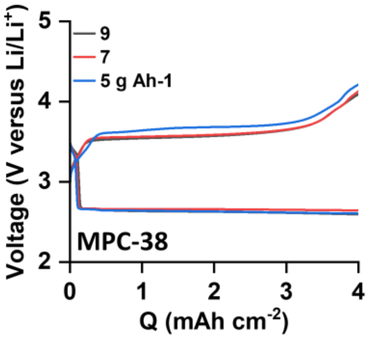

Figure S26. First cycle voltage profiles of MPC-38 electrode cycled with a fixed capacity of 4 mAh cm^{-2} and different electrolyte loading amounts.

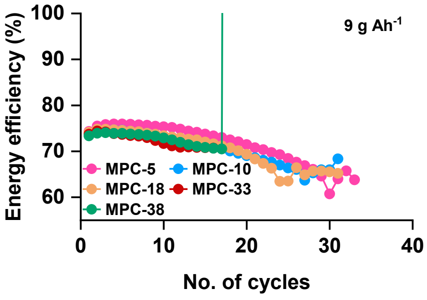

Figure S27. Comparison of energy efficiency over the cycles of different electrodes under 9 g Ah^{-1} electrolyte loading.

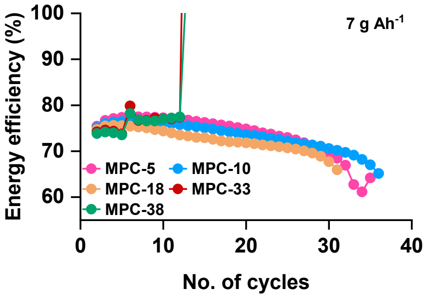

Figure S28. Comparison of energy efficiency over the cycles of different electrodes under 7 g Ah^{-1} electrolyte loading.

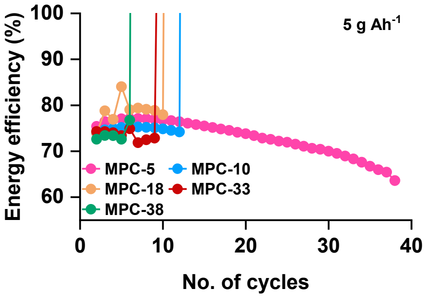

Figure S29. Comparison of energy efficiency over the cycles of different electrodes under 5 g Ah^{-1} electrolyte loading.

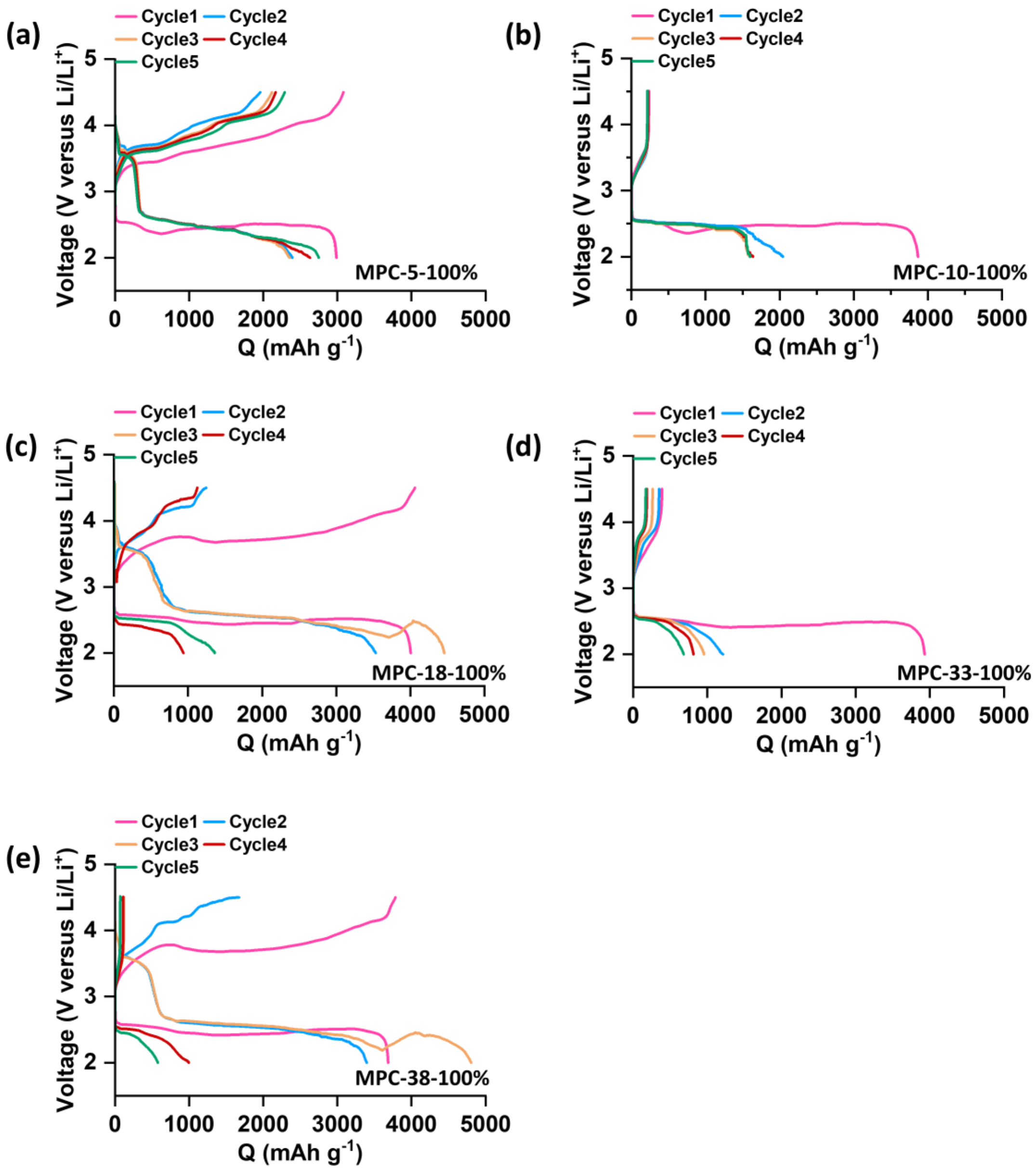

Figure S30. Discharge/charge voltage profiles of different cells with 100% electrolyte loading in the voltage range of 2.0-4.5 V versus Li/Li^{+}.

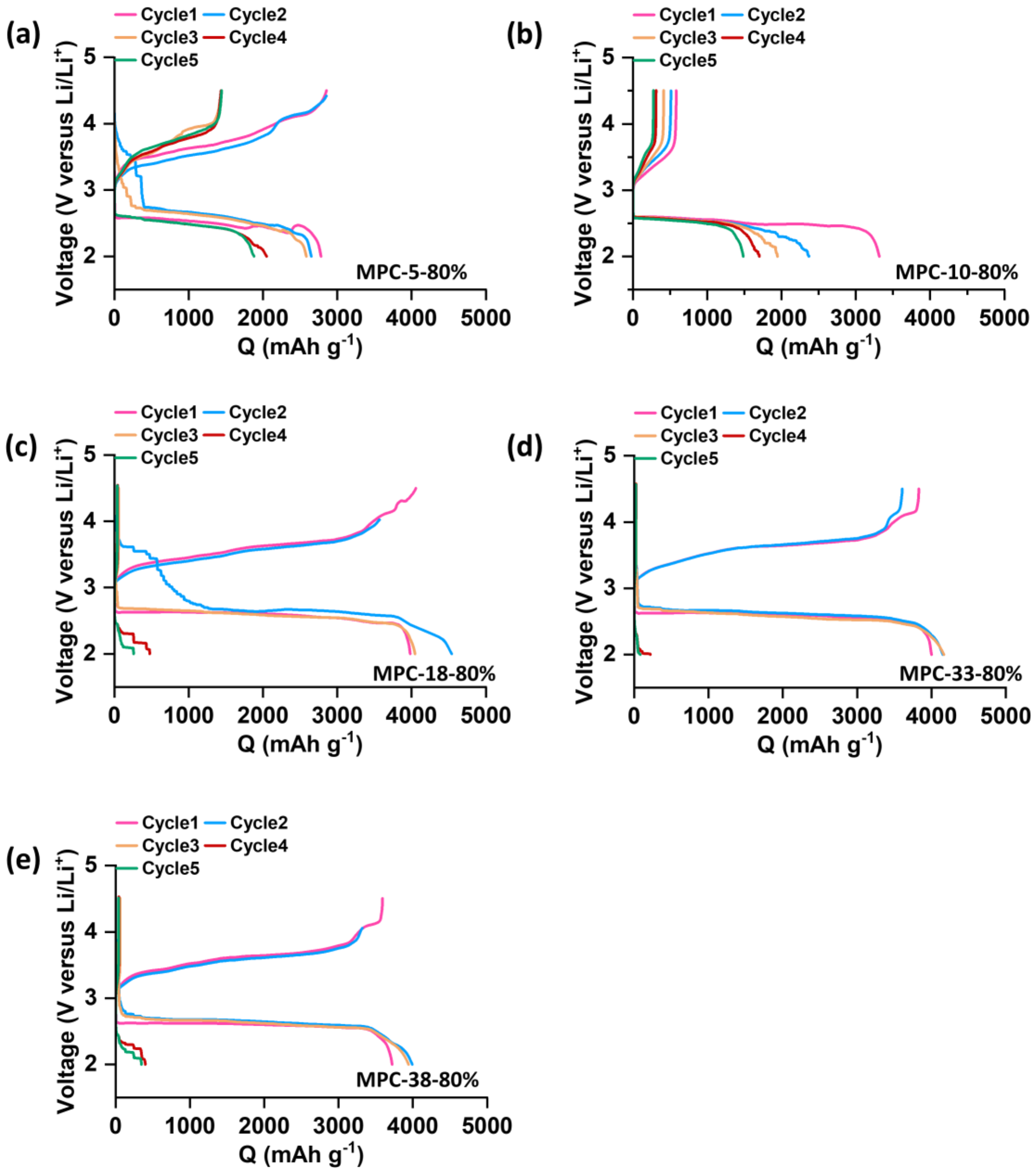

Figure S31. Discharge/charge voltage profiles of different cells with 80% electrolyte loading in the voltage range of 2.0-4.5 V versus Li/Li^{+}.

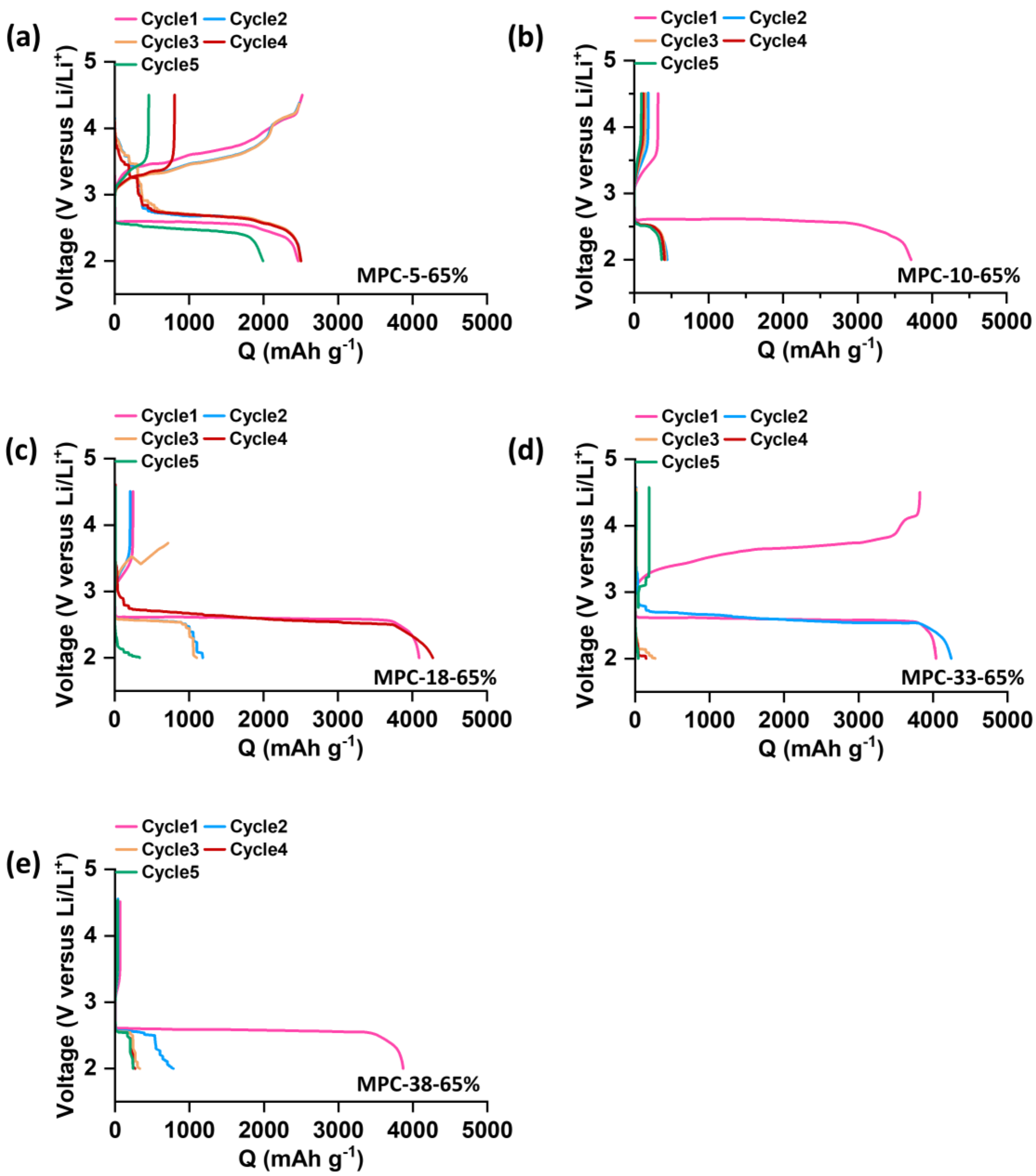

Figure S32. Discharge/charge voltage profiles of different cells with 65% electrolyte loading in the voltage range of 2.0-4.5 V versus Li/Li^{+}.

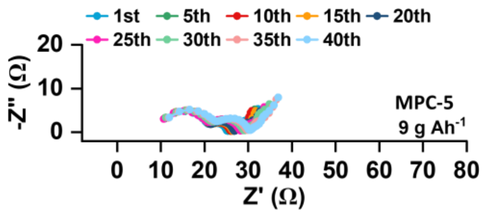

Figure 33. Nyquist plots of the LOB cells with MPC-5 after specific cycles (charged state) with a limited capacity of 4 mAh cm^{-2} under 9 g Ah^{-1} electrolyte loading amount.

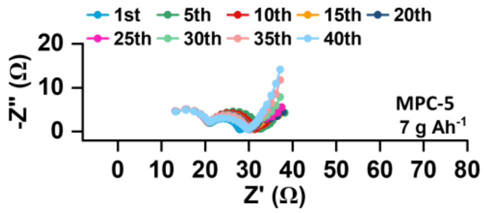

Figure 34. Nyquist plots of the LOB cells with MPC-5 after specific cycles (charged state) with a limited capacity of 4 mAh cm^{-2} under 7 g Ah^{-1} electrolyte loading amount.

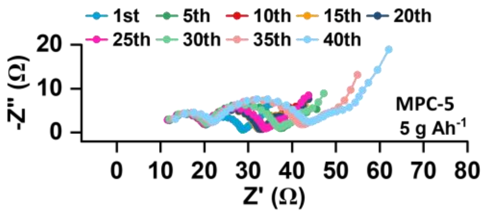

Figure 35. Nyquist plots of the LOB cells with MPC-5 after specific cycles (charged state) with a limited capacity of 4 mAh cm^{-2} under 5 g Ah^{-1} electrolyte loading amount.

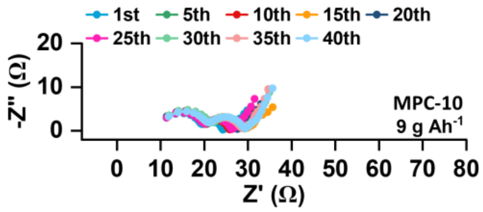

Figure 36. Nyquist plots of the LOB cells with MPC-10 after specific cycles (charged state) with a limited capacity of 4 mAh cm^{-2} under 9 g Ah^{-1} electrolyte loading amount.

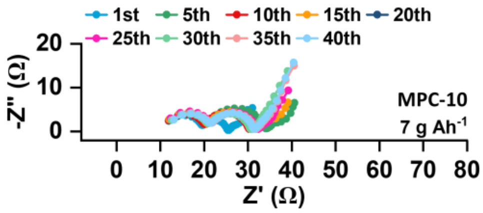

Figure 37. Nyquist plots of the LOB cells with MPC-10 after specific cycles (charged state) with a limited capacity of 4 mAh cm^{-2} under 7 g Ah^{-1} electrolyte loading amount.

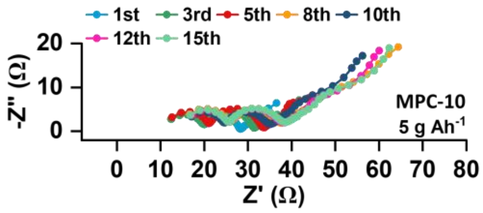

Figure 38. Nyquist plots of the LOB cells with MPC-10 after specific cycles (charged state) with a limited capacity of 4 mAh cm^{-2} under 5 g Ah^{-1} electrolyte loading amount.

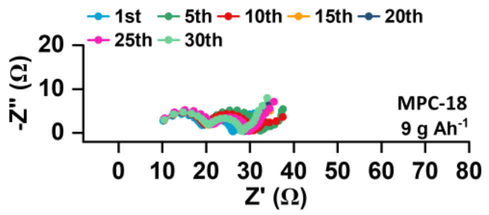

Figure 39. Nyquist plots of the LOB cells with MPC-18 after specific cycles (charged state) with a limited capacity of 4 mAh cm^{-2} under 9 g Ah^{-1} electrolyte loading amount.

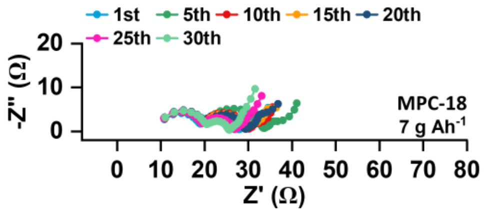

Figure 40. Nyquist plots of the LOB cells with MPC-18 after specific cycles (charged state) with a limited capacity of 4 mAh cm^{-2} under 7 g Ah^{-1} electrolyte loading amount.

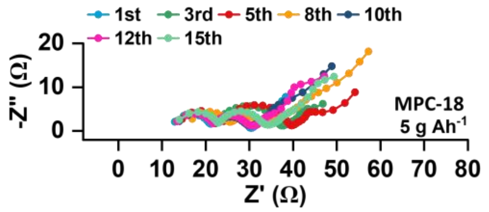

Figure 41. Nyquist plots of the LOB cells with MPC-18 after specific cycles (charged state) with a limited capacity of 4 mAh cm^{-2} under 5 g Ah^{-1} electrolyte loading amount.

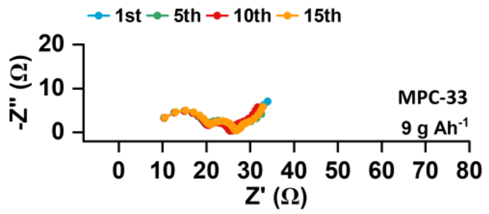

Figure 42. Nyquist plots of the LOB cells with MPC-33 after specific cycles (charged state) with a limited capacity of 4 mAh cm^{-2} under 9 g Ah^{-1} electrolyte loading amount.

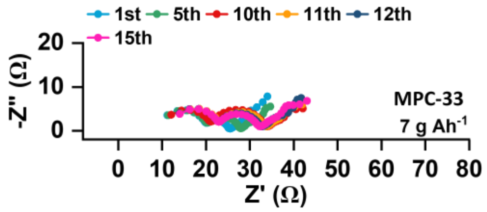

Figure 43. Nyquist plots of the LOB cells with MPC-33 after specific cycles (charged state) with a limited capacity of 4 mAh cm^{-2} under 7 g Ah^{-1} electrolyte loading amount.

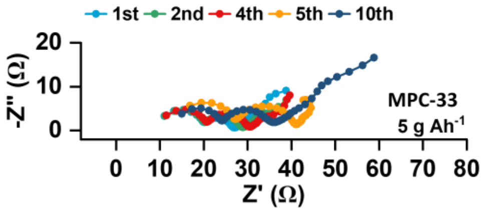

Figure 44. Nyquist plots of the LOB cells with MPC-33 after specific cycles (charged state) with a limited capacity of 4 mAh cm^{-2} under 5 g Ah^{-1} electrolyte loading amount.

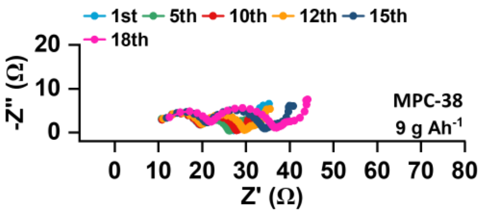

Figure 45. Nyquist plots of the LOB cells with MPC-38 after specific cycles (charged state) with a limited capacity of 4 mAh cm^{-2} under 9 g Ah^{-1} electrolyte loading amount.

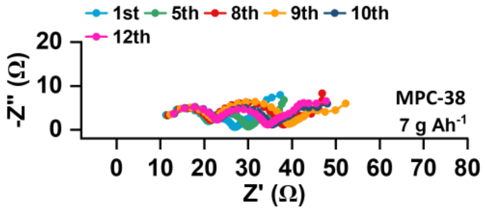

Figure 46. Nyquist plots of the LOB cells with MPC-38 after specific cycles (charged state) with a limited capacity of 4 mAh cm^{-2} under 7 g Ah^{-1} electrolyte loading amount.

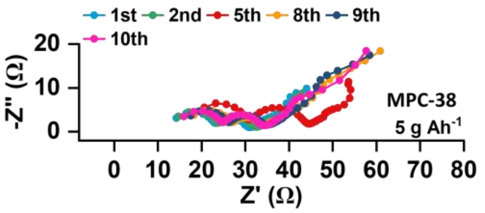

Figure 47. Nyquist plots of the LOB cells with MPC-38 after specific cycles (charged state) with a limited capacity of 4 mAh cm^{-2} under 5 g Ah^{-1} electrolyte loading amount.

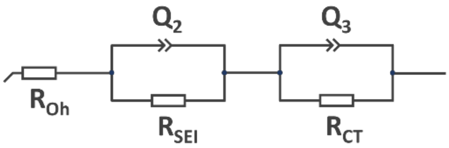

Figure S48. The equivalent circuit used to fit the Nyquist plots.

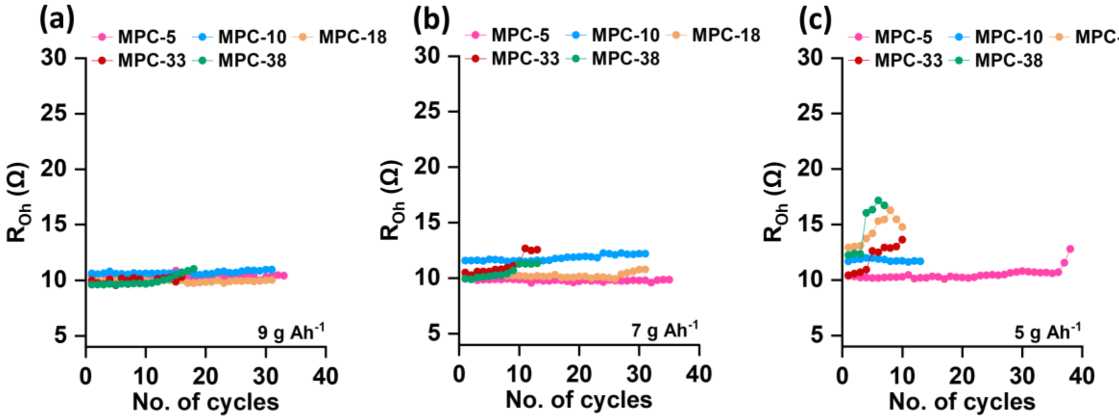

Figure S49. R_{Oh} values of the LOB cells with different electrodes under (a) 9 g Ah^{-1}, (b) 7 g Ah^{-1}, and (c) 5 g Ah^{-1} electrolyte loading amounts.

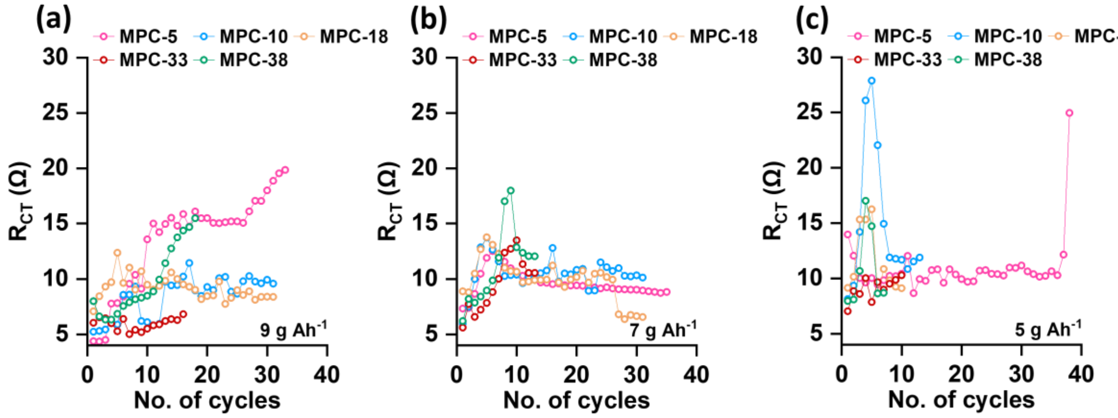

Figure S50. R_{CT} values of different electrodes under (a) 9 g Ah^{-1}, (b) 7 g Ah^{-1}, and (c) 5 g Ah^{-1} electrolyte loading amounts.

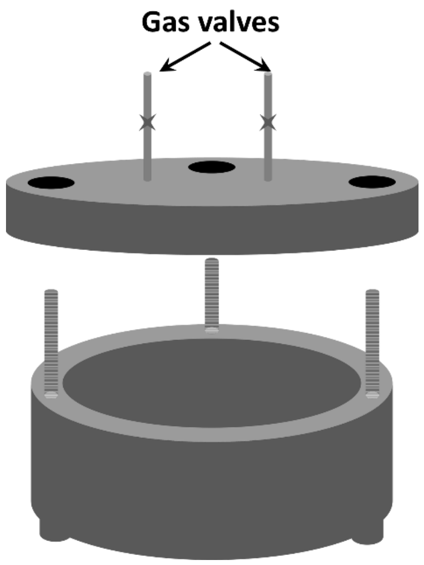

Figure S51. Schematic representation of the flow-type cell used for the online MS analysis.

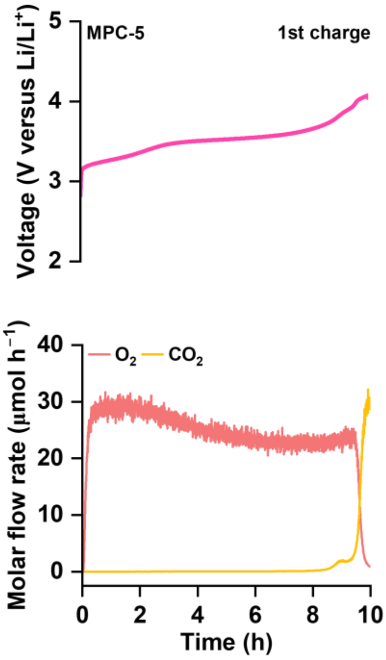

Figure S52. Voltage profile and the corresponding O_2 and CO_2 evolution rates for the first charge of MPC-5.

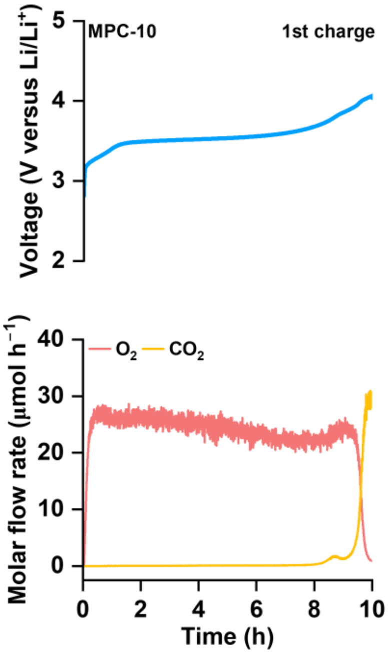

Figure S53. Voltage profile and the corresponding O_2 and CO_2 evolution rates for the first charge of MPC-10.

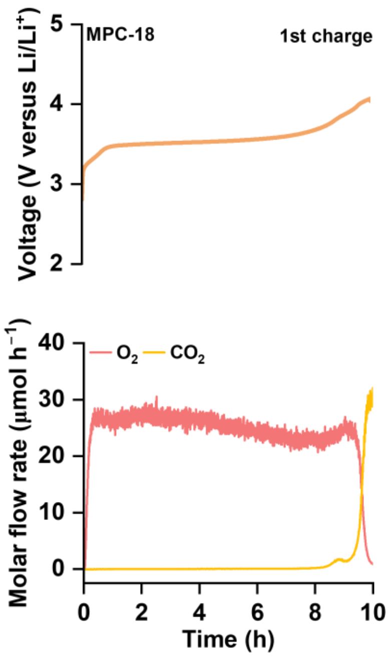

Figure S54. Voltage profile and the corresponding O_2 and CO_2 evolution rates for the first charge of MPC-18.

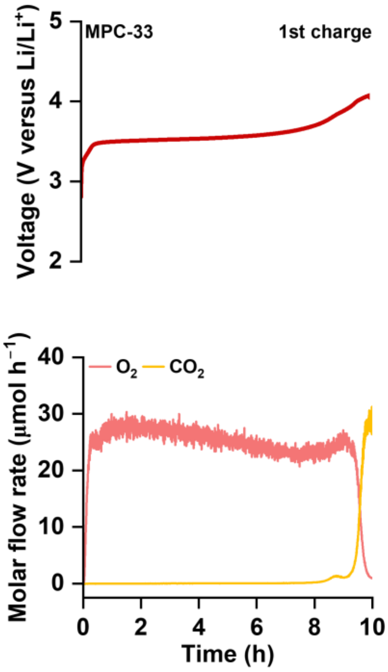

Figure S55. Voltage profile and the corresponding O_2 and CO_2 evolution rates for the first charge of MPC-33.

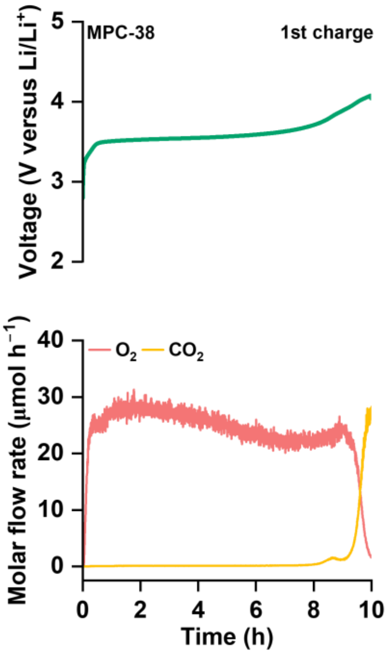

Figure S56. Voltage profile and the corresponding O_2 and CO_2 evolution rates for the first charge of MPC-38.

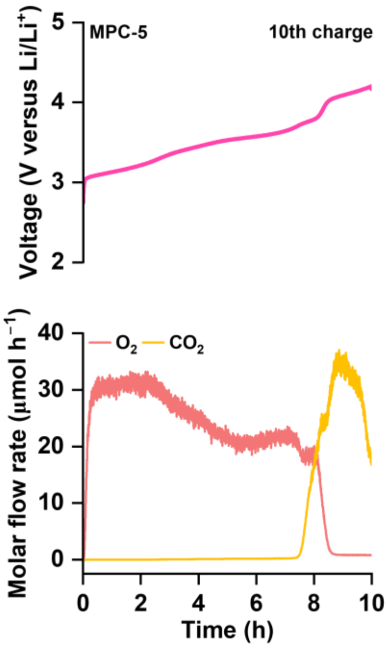

Figure S57. Voltage profile and the corresponding O_2 and CO_2 evolution rates for the 10th charge of MPC-5.

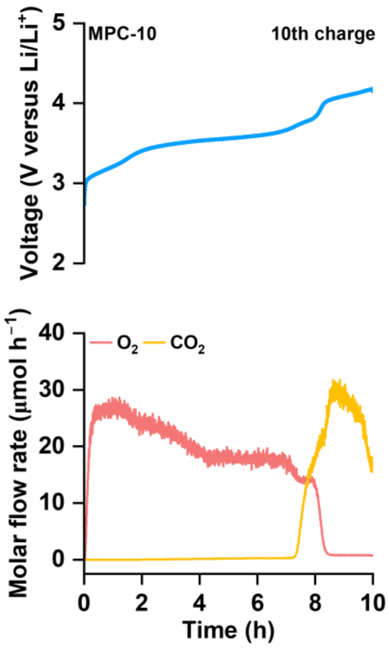

Figure S58. Voltage profile and the corresponding O_2 and CO_2 evolution rates for the 10th charge of MPC-10.

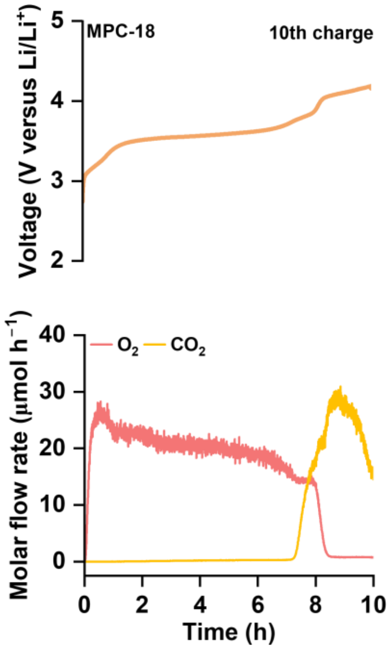

Figure S59. Voltage profile and the corresponding O_2 and CO_2 evolution rates for the 10th charge of MPC-18.

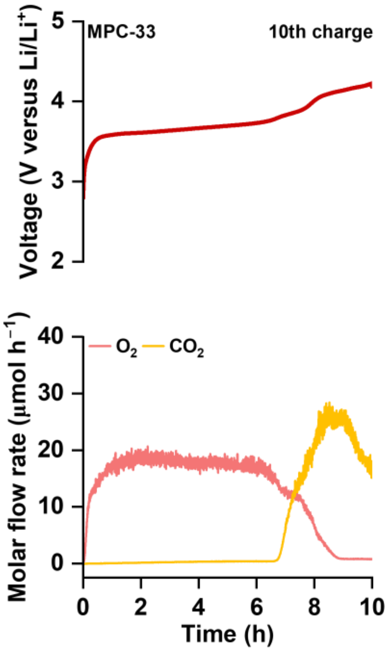

Figure S60. Voltage profile and the corresponding O_2 and CO_2 evolution rates for the 10th charge of MPC-33.

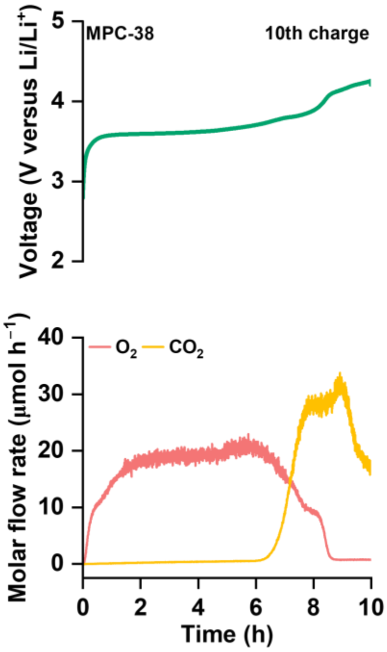

Figure S61. Voltage profile and the corresponding O_2 and CO_2 evolution rates for the 10th charge of MPC-38.

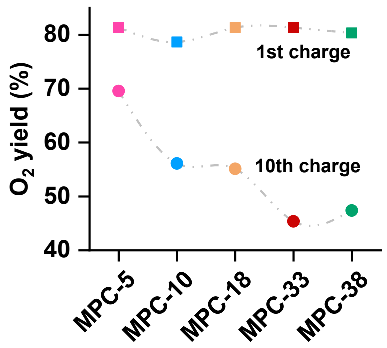

Figure S62. Comparison of O_2 yield of different electrodes at the first and 10th charge.

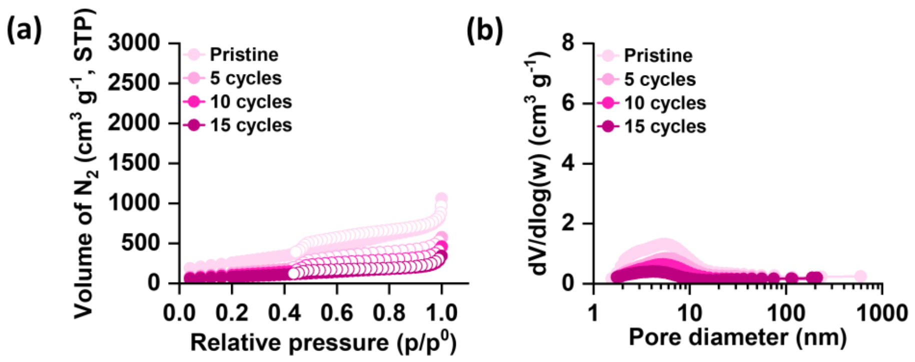

Figure S63. N_2 adsorption/desorption isotherms (a) and BJH pore size distribution curves (b) of MPC-5 electrode at different stages of cycling.

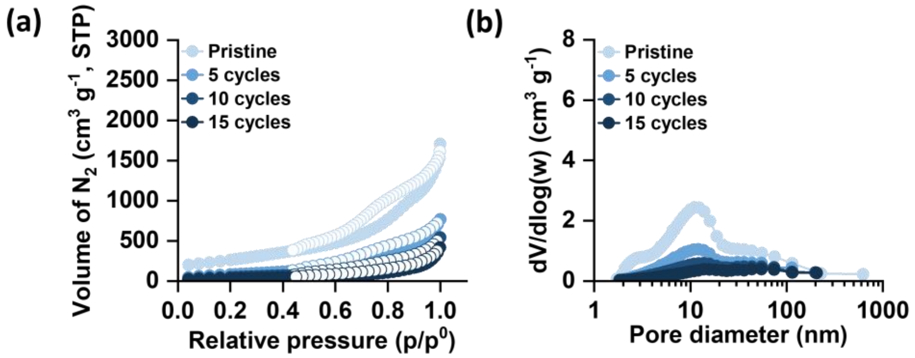

Figure S64. N_2 adsorption/desorption isotherms (a) and BJH pore size distribution curves (b) of MPC-10 electrode at different stages of cycling.

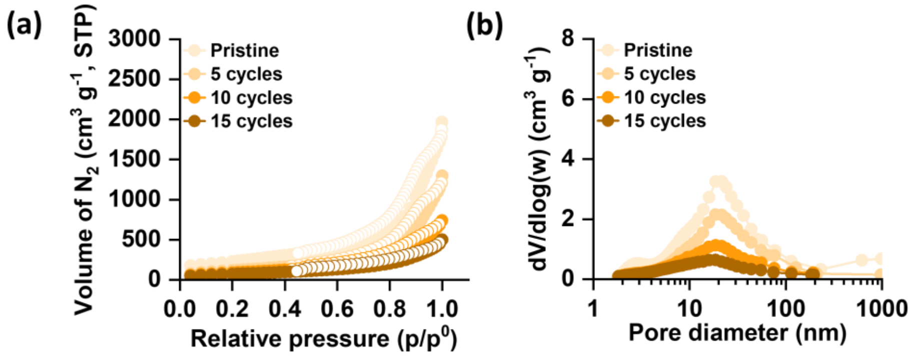

Figure S65. N_2 adsorption/desorption isotherms (a) and BJH pore size distribution curves (b) of MPC-18 electrode at different stages of cycling.

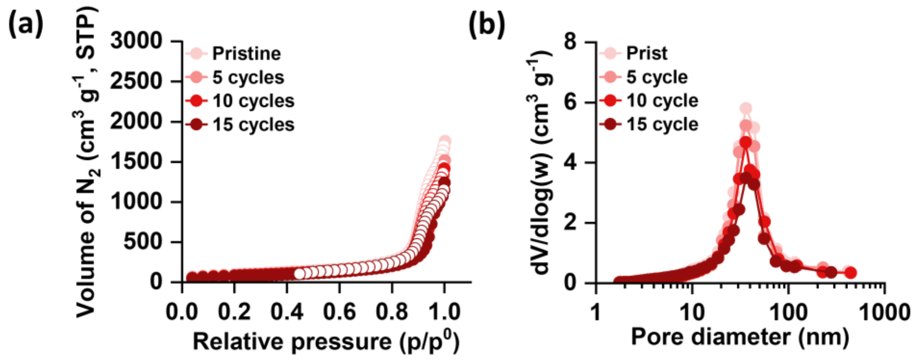

Figure S66. N_2 adsorption/desorption isotherms (a) and BJH pore size distribution curves (b) of MPC-33 electrode at different stages of cycling.

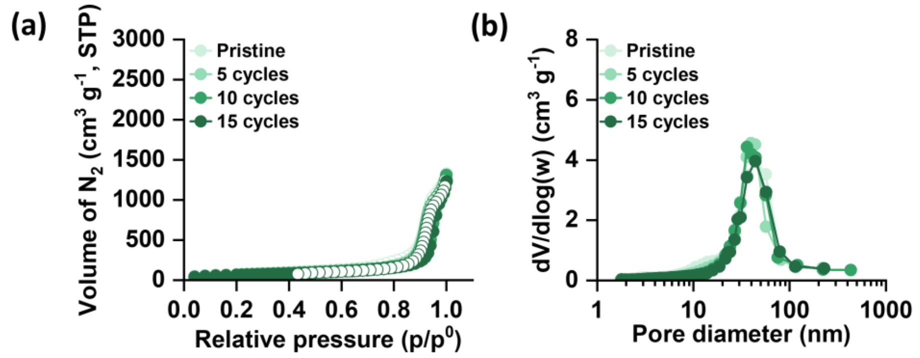

Figure S67. N_2 adsorption/desorption isotherms (a) and BJH pore size distribution curves (b) of MPC-38 electrode at different stages of cycling.

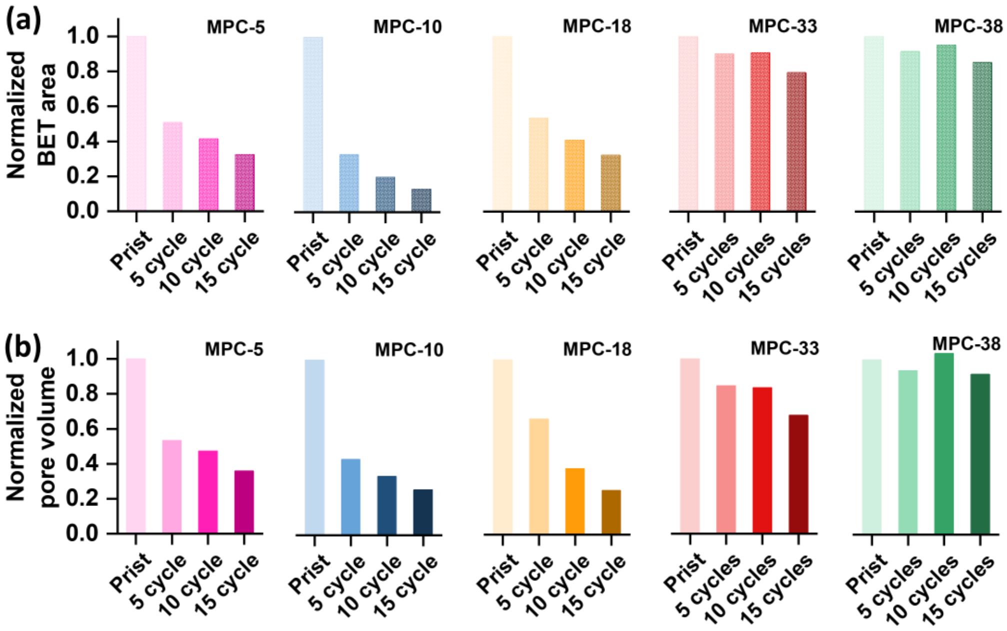

Figure S68. Comparison of BET surface area (a) and pore volume (b) changes, normalized to the values of the pristine electrodes, after specific cycles

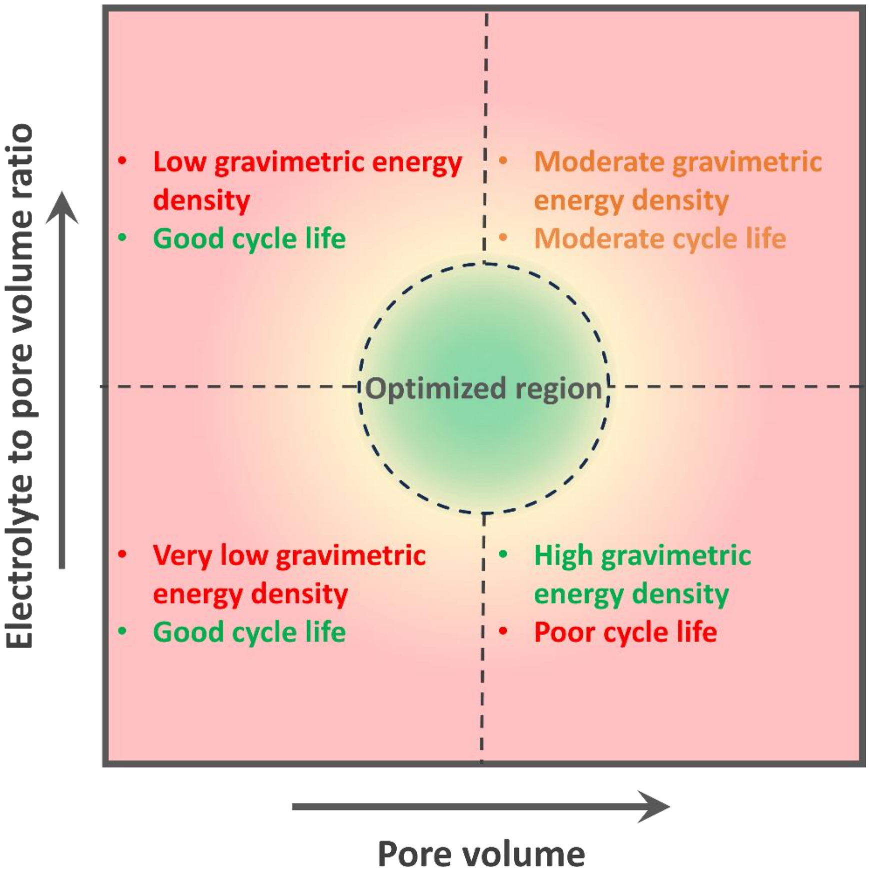

Figure S69. A schematic summary on the role of optimization of electrode pore volume and electrolyte loading amounts to balance the gravimetric energy density and cycling stability of LOBs.

Table S1. BET surface area and pore volume of different carbon powders measured by N_2 adsorption/desorption measurements.

Carbon sample	Pore diameter (nm)	BET surface area ($m^2\ g^{-1}$)	Pore volume ($cm^3\ g^{-1}$)
MPC-5	5	1423	2.0
MPC-10	10	1233	2.98
MPC-18	18	1117	3.71
MPC-33	33	757	3.93
MPC-38	38	623	3.04

Table S2. Masses of different cell components (2 cm × 2 cm pouch cell) used for the calculation of gravimetric energy densities of the cells in Figure 4d,e, Figure S18, and Table S3.

Sample	Carbon loading (g)	EL loading (%)	Mass of EL (g)	Mass of separator (g)	EL in separator (g)	GDL (g)	Li (g)	Current collector (g)
MPC-5	0.024	100	0.134	0.00432	0.00356	0.0052	0.01068	0.0072
MPC-10	0.0188		0.115	0.00432	0.00356	0.0052	0.01068	0.0072
MPC-18	0.0193		0.13	0.00432	0.00356	0.0052	0.01068	0.0072
MPC-33	0.0195		0.137	0.00432	0.00356	0.0052	0.01068	0.0072
MPC-38	0.0209		0.143	0.00432	0.00356	0.0052	0.01068	0.0072
MPC-5	0.0239	80	4.48	0.00432	0.00356	0.0052	0.01068	0.0072
MPC-10	0.0187		4.88	0.00432	0.00356	0.0052	0.01068	0.0072
MPC-18	0.0191		5.39	0.00432	0.00356	0.0052	0.01068	0.0072
MPC-33	0.0189		5.61	0.00432	0.00356	0.0052	0.01068	0.0072
MPC-38	0.0199		5.43	0.00432	0.00356	0.0052	0.01068	0.0072
MPC-5	0.0237	65	3.62	0.00432	0.00356	0.0052	0.01068	0.0072
MPC-10	0.0185		3.96	0.00432	0.00356	0.0052	0.01068	0.0072
MPC-18	0.0188		4.38	0.00432	0.00356	0.0052	0.01068	0.0072
MPC-33	0.0187		4.56	0.00432	0.00356	0.0052	0.01068	0.0072
MPC-38	0.0198		4.44	0.00432	0.00356	0.0052	0.01068	0.0072

Table S3: Parameters used for the calculation of specific capacities and gravimetric energy densities of the cells with various electrodes and different levels of electrolyte loading.

Sample	Mass loading of C (g)	EL loading (%)	EL to carbon mass ratio	Q (Normalized to carbon) (mAh g^{-1})	Q (Normalized to carbon + EL) (mAh g^{-1})	Average discharge voltage (V)	Gravimetric energy density (Wh kg^{-1})
MPC-5	0.024	100	5.58	2987	454	2.46	933
MPC-10	0.0188		6.12	3866	543	2.46	1085
MPC-18	0.0193		6.74	4004	518	2.49	1067
MPC-33	0.0195		7.03	3928	489	2.46	1005
MPC-38	0.0209		6.84	3685	470	2.48	980
MPC-5	0.0239	80	4.48	2780	508	2.49	1022
MPC-10	0.0187		4.88	3874	658	2.51	1290
MPC-18	0.0191		5.39	3981	623	2.57	1276
MPC-33	0.0189		5.61	3999	605	2.58	1251
MPC-38	0.0199		5.43	3721	579	2.58	1202
MPC-5	0.0237	65	3.62	2465	533	2.53	1052
MPC-10	0.0185		3.96	3716	749	2.57	1439
MPC-18	0.0188		4.38	4089	760	2.58	1500
MPC-33	0.0187		4.56	4039	726	2.58	1443
MPC-38	0.0198		4.44	3869	711	2.56	1413

Table S4. Comparison of different cell cycle parameters of the LOB cells shown in Figure 4e.

Serial no.	Areal capacity ($mAh\ cm^{-2}$)	Average discharge voltage (V)	Areal mass ($mg\ cm^{-2}$)	Gravimetric energy density ($Wh\ kg^{-1}$)	Reference
1	17.92	2.46	47.24	933	MPC-5-100%
2	18.17	2.46	41.19	1085	MPC-10-100%
3	19.31	2.49	45.06	1067	MPC-18-100%
4	19.15	2.46	46.86	1005	MPC-33-100%
5	19.25	2.48	48.71	980	MPC-38-100%
6	16.61	2.49	40.46	1022	MPC-5-80%
7	18.11	2.51	35.24	1290	MPC-10-80%
8	19.00	2.57	38.26	1276	MPC-18-80%
9	18.89	2.58	38.96	1251	MPC-33-80%
10	18.51	2.58	39.71	1202	MPC-38-80%
11	14.60	2.53	35.11	1052	MPC-5-65%
12	17.18	2.57	30.69	1439	MPC-10-65%
13	19.21	2.58	33.04	1500	MPC-18-65%
14	18.88	2.58	33.74	1443	MPC-33-65%
15	19.15	2.56	34.69	1413	MPC-38-65%
16	30.8	2.65	103	793	[2]
17	3	2.69	14.85	543	[3]
18	4	2.72	63.26	393	[3]
19	12.6	2.5	80	393	[4]
20	1.2	2.5	80	37.5	[4]
21	10	2.7	138.09	195	[5]
22	3.55	2.65	7.75	1214	[16]
23	3.38	2.75	89.84	103	[17]
24	6.7	2.75	89.84	205	[17]
25	9.74	2.75	89.84	298	[17]
26	13.45	2.75	89.84	411	[17]

References

[1] J. Saengkaew, T. Kameda, M. Ono, S. Matsuda, Self-Standing Porous Carbon Electrodes for Lithium–Oxygen Batteries Under Lean Electrolyte and High Areal Capacity Conditions. *Mater. Adv.* **2022**, *3*, 3536.

[2] W. Yu, Z. Shen, T. Yoshii, S. Iwamura, M. Ono, S. Matsuda, M. Aoki, T. Kondo, S. R. Mukai, S. Nakanishi, H. Nishihara, Hierarchically Porous and Minimally Stacked Graphene Cathodes for High-Performance Lithium–Oxygen Batteries. *Adv. Energy Mater.* **2024**, *14*, 2303055.

[3] S. Matsuda, E. Yasukawa, T. Kameda, S. Kimura, S. Yamaguchi, Y. Kubo, K. Uosaki, Carbon-Black-Based Self-Standing Porous Electrode for 500 Wh/Kg Rechargeable Lithium-Oxygen Batteries. *Cell. Rep. Phys. Sci.* **2021**, *2*, 100506.

[4] W. Chen, W. Yin, Y. Shen, Z. Huang, X. Li, F. Wang, W. Zhang, Z. Deng, Z. Zhang, Y. Huang, High Areal Capacity, Long Cycle Life Li-O_2 Cathode Based on Highly Elastic Gel Granules. *Nano Energy* **2018**, *47*, 353.

[5] Y. J. Lee, S. H. Park, S. H. Kim, Y. Ko, K. Kang, Y. J. Lee, High-Rate and High-Areal-Capacity Air Cathodes with Enhanced Cycle Life Based on RuO_2/MnO_2 Bifunctional Electrocatalysts Supported on CNT for Pragmatic Li–O_2 Batteries. *ACS Catal.* **2018**, *8*, 2923.

[6] M. Jenkins, D. Dewar, M. Lagnoni, S. Yang, G. J. Rees, A. Bertei, L. R. Johnson, X. Gao, P. G. Bruce, A High Capacity Gas Diffusion Electrode for Li–O_2 Batteries. *Adv. Mater.* **2024**, *36*, 2405715.

[7] S. H. Park, Y. J. Cheon, Y. J. Lee, K. H. Shin, Y. Y. Hwang, Y. S. Jeong, Y. J. Lee, Maximal Utilization of a High-Loading Cathode in Li–O_2 Batteries: A Double Oxygen Supply System. *ACS Appl. Mater. Interfaces* **2019**, *11*, 30872.

[8] H. Song, S. Xu, Y. Li, J. Dai, A. Gong, M. Zhu, C. Zhu, C. Chen, Y. Chen, Y. Yao, B. Liu, J. Song, G. Pastel, L. Hu, Hierarchically Porous, Ultrathick, “Breathable” Wood-Derived Cathode for Lithium-Oxygen Batteries. *Adv. Energy Mater.* **2018**, *8*, 1701203.

[9] X. Gao, Y. Chen, L. Johnson, P. G. Bruce, Promoting Solution Phase Discharge in Li–O_2 Batteries Containing Weakly Solvating Electrolyte Solutions. *Nat. Mater.* **2016**, *15*, 882.

[10] C. Chen, S. Xu, Y. Kuang, W. Gan, J. Song, G. Chen, G. Pastel, B. Liu, Y. Li, H. Huang, L. Hu, Nature-Inspired Tri-Pathway Design Enabling High-Performance Flexible Li–O_2 Batteries *Adv. Energy Mater.* **2019**, *9*, 1802964.

[11] J. Zhang, B. Sun, Y. Zhao, A. Tkacheva, Z. Liu, K. Yan, X. Guo, A. M. McDonagh, D. Shanmukaraj, C. Wang, T. Rojo, M. Armand, Z. Peng, G. Wang, A Versatile Functionalized Ionic Liquid to Boost the Solution-Mediated Performances of Lithium-Oxygen Batteries. *Nat. Commun.* **2019**, *10*, 602.

[12] X. Gao, Y. Chen, L. R. Johnson, Z. P. Jovanov, P. G. Bruce, A Rechargeable Lithium–Oxygen Battery with Dual Mediators Stabilizing the Carbon Cathode. *Nat. Energy* **2017**, *2*, 17118.

[13] C. Zhu, L. Du, J. Luo, H. Tang, Z. Cui, H. Song, S. Liao, A Renewable Wood-Derived Cathode for Li–O_2 Batteries. *J. Mater. Chem. A* **2018**, *6*, 14291.

[14] W.-B. Luo, S.-L. Chou, J.-Z. Wang, Y.-C. Zhai, H.-K. Liu, A Metal-Free, Free-Standing, Macroporous Graphene@g-C_3N_4 Composite Air Electrode for High-Energy Lithium Oxygen Batteries. *Small* **2015**, *11*, 2817.

[15] Y. Lin, B. Moitoso, C. Martinez-Martinez, E. D. Walsh, S. D. Lacey, J.-W. Kim, L. Dai, L. Hu, J. W. Connell, Ultrahigh-Capacity Lithium–Oxygen Batteries Enabled by Dry-Pressed Holey Graphene Air Cathodes. *Nano Lett.* **2017**, *17*, 3252.

[16] H. C. Lee, J. O. Park, M. Kim, H. J. Kwon, J.-H. Kim, K. H. Choi, K. Kim, D. Im, High-Energy-Density Li-O_2 Battery at Cell Scale with Folded Cell Structure. *Joule* **2019**, *3*, 542.

[17] S. Zhao, L. Zhang, G. Zhang, H. Sun, J. Yang, S. Lu, Failure Analysis of Pouch-Type Li–O_2 Batteries with Superior Energy Density. *J. Energy Chem.* **2020**, *45*, 74.
